# The Beneficial Impact of the Black Chokeberry Extract against the Oxidative Stress in the Sublingual Salivary Gland of Rats Intoxicated with Cadmium

**DOI:** 10.1155/2021/6622245

**Published:** 2021-12-31

**Authors:** Barbara M. Onopiuk, Zofia N. Dąbrowska, Joanna Rogalska, Malgorzata M. Brzóska, Adam Dąbrowski, Kamil Bijowski, Pawel Onopiuk, Barbara Mroczko, Karolina Orywal, Ewa Dąbrowska

**Affiliations:** ^1^Private Dental Office in Bialystok, Bialystok 15-773, Poland; ^2^Department of Periodontal and Oral Mucosa Diseases, Medical University of Bialystok, Bialystok 15-276, Poland; ^3^Department of Toxicology, Medical University of Bialystok, Bialystok 15-222, Poland; ^4^Department of Maxillofacial and Plastic Surgery, Medical University of Bialystok, Bialystok 15-276, Poland; ^5^Department of Otolaryngology, Medical University of Bialystok, Bialystok 15-276, Poland; ^6^Department of Biochemical Diagnostics, Medical University of Bialystok, Bialystok 15-269, Poland; ^7^Department of Gerostomatology, Medical University of Bialystok, Bialystok 15-286, Poland

## Abstract

Cadmium (Cd) is one of the most harmful xenobiotics to which humans are exposed, mainly by the oral route, throughout life. Preventive strategies are searched as low intoxication with this element, among others due to its prooxidative properties, can be deleterious to health and the exposure to it is continuously increasing. Recently, interest has been paid to plant raw materials with a high antioxidative potential to oppose the prooxidative properties of cadmium, such as black chokeberry (*Aronia melanocarpa* L. fruit), which is rich in polyphenolic compounds. The study was aimed at assessing whether the chokeberry extract may counteract the prooxidative impact of low-level and moderate repeated intoxication with cadmium on the sublingual salivary gland. The investigation was performed on 96 Wistar rats (females), which were treated with a 0.1% aqueous extract from chokeberries or/and a diet containing 1 or 5 mg Cd/kg for 3 and 10 months, and control animals. The intoxication with cadmium, in a dose- and time-dependent manner, attenuated the enzymatic and nonenzymatic antioxidative potential and increased the concentration of hydrogen peroxide and total oxidative status of the sublingual salivary gland resulting in an occurrence of oxidative stress, enhancement of lipid peroxidation, and oxidative injuries of proteins in this salivary gland. The treatment with the black chokeberry extract during the intoxication with cadmium prevented this xenobiotic-caused oxidative/reductive imbalance and oxidative modifications of proteins and lipids in the salivary gland. The above results allow the conclusion that the consumption of black chokeberry products during intoxication with cadmium can prevent oxidative stress and its consequences in the sublingual salivary gland and thus counteract the unfavourable impact of this xenobiotic on the oral cavity.

## 1. Introduction

Oxidative stress is a state of disturbed balance between oxidants and antioxidants with a predominance of the former ones, which leads to a disruption of redox signalling and control and/or oxidative damage to cellular molecules (e.g., lipids, proteins, and nucleic acids) [1, 2]. This state is involved in the development of numerous disorders, including these affecting the oral cavity [2–4]. The oral cavity is the place where reactive oxygen species (ROS) are produced under the influence of many various factors with a prooxidative potential, and the main source of ROS is periodontitis associated with inflammation of the gums and jaw bone [3, 4]. The contribution of oxidative stress in the aetiology of periodontal diseases, gingivitis, osteitis, xerostomia, Sjögren syndrome, oral lichen planus, aphthous stomatitis, oral leucoplakia, and oral cancer has been revealed [2, 3]. The occurrence of the state of destroyed balance between antioxidants and oxidants is one of the mechanisms of toxic action of some xenobiotics on the oral cavity, including medications or heavy metals, e.g., cadmium (Cd) [4–10].

Cadmium is a toxic heavy metal to which inhabitants of highly industrialized countries are exposed throughout their lifetime [11–14]. Pollution of the natural environment with this xenobiotic from anthropogenic sources (e.g., factories, metal smelters, or power plants) has elevated in the past decades, creating a real threat to all biological ecosystems and human health [11, 12]. The widespread presence of cadmium in the environment is a cause of food and drinking water pollution, which is the main source of the general population's exposure to this toxic heavy metal [13–15]. Moreover, tobacco smoke is also a source of the intake of cadmium [16–18].

With continuous exposure, cadmium is accumulated in various tissues and organs, where it exerts toxic action [19, 20]. Available data indicate that moderate and even relatively low intoxication with this xenobiotic may pose a threat for health [11–14, 21–25]. Cadmium destroys the function of the renal tubules and liver, leads to bone tissue demineralization, and induces hypertension [11, 21–24]. Moreover, the previous results of our own in vivo studies conducted in animal models show that repeated exposure to this xenobiotic can have a damaging impact on the organs and tissues located within the oral cavity [7–9]; however, so far this effect has not been studied in humans. The unfavourable influence may result, among others, from prooxidative properties of cadmium that lead to oxidative injuries within the oral cavity, including the salivary glands [7–9, 26–28].

Taking into account the fact that already relatively low cadmium intoxication may be threatening to human health and that exposure to this metal is still on the increase [11–14, 21–25], effective strategies are searched to limit the toxic action of this element [11, 14, 20, 29–31]. An object of special interest in this regard are products of natural origin, such as plants abundant in polyphenols and characterized by high antioxidative potential [11, 14, 20, 29–32]. The black chokeberries (berries of *Aronia melanocarpa* L. and *A. melanocarpa* (Michx.) Elliott, *Rosaceae*) are characterized by a very high content of these compounds, especially proanthocyanidins and anthocyanins [29, 32, 33], and thus, they exhibit numerous beneficial actions for health, such as anti-inflammatory, antidiabetic, antiatherosclerotic, antithrombotic, and antineoplastic effects [29, 34–36]. The beneficial activity of polyphenols in a variety of diseases and other states arises from the strong antioxidative properties of these compounds determined by their structure and the location of hydroxyl groups (–OH) in the aromatic ring [29, 32, 33]. Moreover, experimental studies carried out by our research team, in an animal model reflecting environmental exposure of the general population to cadmium, have shown that the chokeberry extract (ChE) prevented the retention of this xenobiotic in the body and some unfavourable health outcomes of chronic exposure to it or at least attenuated them partially [7, 8, 19, 26–28, 37]. It has been revealed in this model that ChE can protect the liver [26, 27], bones [28, 37], and the parotid and submandibular salivary glands [7, 8] against oxidative stress induced by cadmium and oxidative changes as a consequence.

In the case of the general population, the oral route is the main way of cadmium intake into the body [11, 13] and animal studies show that this xenobiotic may induce pathological changes in the salivary glands, resulting among others from oxidative stress [7–9]. Moreover, other factors, to which individuals may be exposed, including tobacco smoke, ethyl alcohol, fluorides, ozone, titanium, laser light, and radiotherapy, as well as high-fat and high-protein diets, increase the amount of ROS in the oral environment [3, 4, 38–42]. Destroying the oxidative/antioxidative balance in the oral cavity causes damage to the components of salivary gland cells and increases chronic local and systemic inflammation (by increasing the production and secretion of proinflammatory cytokines) and leads to dysfunction of the salivary glands, which can be seen in the form of changes in both the quantity and quality of salivary secretion. Oxidative stress affects the functioning of the salivary glands changing, as a result, the composition, properties, and flow rate of the saliva [3, 4, 38, 40, 41, 43].

Although the molecular mechanisms explaining the role of oxidative stress in the development of oral diseases are not well established, it is known that an increase in the amounts of free radicals (FR) and ROS and attenuation of the antioxidative capacity are involved in the development of oral pathologies [3, 4, 38, 40, 41, 43]. Thus, it is very important to recognize the risk of damage to the various tissues of the oral cavity by prooxidants, including cadmium, as well as to find an efficient method of prevention against the action of these factors. Taking into consideration the available data indicating that already low intoxication with cadmium may induce oxidative stress, as well as the antioxidative impact of ChE under exposure to this xenobiotic [7, 8, 26–28], including especially our findings regarding the parotid and submandibular salivary glands [7, 8], we put forward a hypothesis that the treatment will also lead to oxidative changes in the sublingual salivary gland tissue and that this extract can protect against these actions of this xenobiotic. The fact of revealing that the intoxication with cadmium variously affected the oxidative/antioxidative balance in different tissues, including the salivary glands, and revealing that ChE administration to the animals intoxicated with cadmium has a favourable impact on the parotid and submandibular salivary glands [7, 8] did not allow us to recognize that it will be true also concerning the sublingual salivary glands. Because of various specific functions of particular salivary glands and differences in the defence mechanisms, including the antioxidative capacity of the secreted saliva, the impact of cadmium and/or ChE may be different. The sublingual salivary glands secrete mixed saliva with a predominance of mucosal saliva while the submandibular and parotid glands secrete saliva with a predominance of serous saliva [44]. It has been shown that sublingual and submandibular saliva, due to lower concentrations of enzymatic and nonenzymatic antioxidants, is characterized by much lower total antioxidative status (TAS) than the parotid saliva [44].

In connection with the above, the current study was aimed at finding out if low and moderate repeated intoxication with cadmium may induce oxidative stress and oxidatively modify the macromolecules in the cells of the sublingual salivary gland tissue and whether the administration of ChE during the exposure may protect against this impact of this xenobiotic. For this purpose, indices of the enzymatic (superoxide dismutase (SOD), catalase (CAT), and glutathione peroxidase (GPx)) and nonenzymatic (reduced glutathione (GSH)) antioxidative barrier, TAS (the main marker of antioxidative capacity), total oxidative status (TOS; the main marker of oxidative status), and hydrogen peroxide (H_2_O_2_), as well as protein carbonyl groups (PC) and lipid peroxides (LPO), as markers of oxidative modifications of proteins and lipids, respectively, were assayed in the sublingual salivary gland. Moreover, the impact of ChE on cadmium accumulation in this gland was estimated.

## 2. Materials and Methods

The current paper presents the results of the study being a continuation and extension of our research aimed at recognizing both the unfavourable influence of cadmium and the possible protective impact of ChE on the organs of the oral cavity [7–9]. However, the present study was conducted on a different organ of the oral cavity, which was the sublingual salivary gland. The experimental model used has been presented in detail elsewhere [7, 19, 28, 37], and thus, in this article, only necessary description is provided.

### 2.1. Experimental Model

The left and the right sublingual salivary glands were harvested during the trial that obtained the approval of the Local Ethics Committee for Animal Experiments in Bialystok (Resolution No. 60/2009).

The study used 3-4-week-old 96 female Wistar rats (Crl: WI (Han)) with the initial body weight of ~50 g. The rats were kept in stainless-steel standard laboratory cages. The breeding conditions were optimal for experimental animals (relative humidity 50 ± 10%, temperature 22 ± 2°C, and 12/12 h light/dark cycle). The rats were randomly assigned into 6 groups containing 16 individuals each. The control animals were given unpolluted water to drink and a standard commercial diet (Labofeed; Animal Feed Manufacturer “Morawski”, Kcynia, Lublin, Poland) throughout the experiment. In the course of the first 3 months of the study, the animals were feed with the Labofeed H diet (breeding diet), and thereafter (from the 4th month), the standard Labofeed B diet (maintenance diet) was applied (the composition of the diets is provided in Table S1 in Supplementary Materials). The mean cadmium concentration in the standard diets was <0.06 mg/kg, whereas this element concentration in drinking water was <0.05 *μ*g Cd/l [19]. The animals from the remaining four groups were intoxicated with cadmium in the diet containing 1 or 5 mg Cd/kg and/or received a 0.1% aqueous ChE as the only drinking fluid for 3 and 10 months (Cd_1_ group, Cd_5_ group, ChE group, Cd_1_+ChE group, and Cd_5_+ChE group). The diets polluted with cadmium were produced by Label Food “Morawski” (Kcynia, Poland) by the addition of cadmium chloride (CdCl_2_ × 2.5 H_2_O) into the ingredients of the Labofeed H and Labofeed B standard diets. The animals had unlimited access to food free of pollutants (control group and ChE group) or containing cadmium (Cd_1_ group, Cd_5_ group, Cd_1_+ChE group, and Cd_5_+ChE group) and drinking fluid—unpolluted water (control group, Cd_1_ group, and Cd_5_ group) or 0.1% aqueous ChE (ChE group, Cd_1_+ChE group, and Cd_5_+ChE group). The 0.1% ChE was prepared from the powdered *A. melanocarpa* extract provided by the Adamed Consumer Healthcare Company (Tuszyn, Poland), which contained 65.74% of polyphenolic compounds, including 18.65% of anthocyanins (Certificate KJ 4/2010; Batch No. M100703).

The daily intakes of food and drinking fluids did not differ between the experimental groups (Table S2 in Supplementary Materials). There were no observable abnormalities in the health status of the animals from particular groups and differences in the body weight gain (the mean body weight gain in the control group during 3 and 10 months of the experiment reached 222.7 g and 369.1 g, respectively) [19].

Cadmium concentration in the diet (1 and 5 mg/kg) was chosen to create the conditions of rats' treatment with this xenobiotic comparable to the current low-level and moderate repeated intoxication of humans in industrialized countries. The determination of markers of exposure to cadmium such as its concentration in the urine and blood of the animals feed with the 1 and 5 mg Cd/kg diet alone or with ChE (0.0852–0.2762 *μ*g/g creatinine and 0.103–0.306 *μ*g/l and 0.2839–0.8197 *μ*g/g creatinine and 0.584–1.332 *μ*g/l, respectively) [19] confirmed that the used experimental model well reflects human lifetime intoxication with this toxic element in industrialized countries [11, 21]. The daily cadmium intake in the case of the feeding with the 1 and 5 mg Cd/kg diet was between 41.45 mg/kg body weight (b.w.) and 84.88 *μ*g/kg b.w. and from 222.35 *μ*g/kg b.w. to 404.76 *μ*g/kg b.w., respectively (Table S3 in Supplementary Materials) [7, 19]. To estimate the development of the unfavourable impact of cadmium over time, all investigated parameters were determined after 3 and 10 months. Because the young organism is the most susceptible to cadmium toxicity, performing the measurements after 3 months allowed us to evaluate the impact of this xenobiotic during the period of intensive growth at a young age. Prolongation of the exposure up to 10 months allowed us to estimate the impact of cadmium during adulthood. After the 10-month study duration, the rats reached the age of about 11 months, which corresponds to the age of around 30 in humans [45].

ChE was used in the form of a 0.1% aqueous solution to reach in the animals the daily intake of polyphenolic compounds markedly higher, but not too high, than the average mean intake of these compounds in the general population. The available literature data show that the intake of polyphenols in humans all over the world, because of differences in dietary behaviour, falls within a relatively wide range of values from about 800 mg/24 h to even above 1700 mg/24 h and can be estimated at 1000 mg/24 h on average (14.29 mg/kg b.w. assuming the average body weight reaching 70 kg) [46–49]. Throughout the experiment, the daily intake of ChE in rats ranged from 78.70 mg/kg b.w. to 153.82 mg/kg b.w. (Table S4 in Supplementary Materials) [7, 19]. The consumption of polyphenolic compounds reached from 51.7 mg/kg b.w. to 104.6 mg/kg b.w. (Table S4 in Supplementary Materials) [7, 19] and was several times higher compared to their average intake in humans. According to the available literature, no evidence of any undesirable or toxic actions of polyphenols, as well as chokeberries and their products in both human and experimental animals, was noted [29, 49]. There were no differences in the mean consumption of ChE and cadmium at particular time points between the groups administered with these factors alone or jointly (Tables S3 and S4 in Supplementary Materials) [7, 19].

Since the unfavourable impact of cadmium was evaluated after 3 and 10 months of exposure, the possible protective influence of ChE against the toxic action of this xenobiotic was also evaluated at these time points. This made it possible to assess the impact of the extract administration during the period of life when the animals are the most susceptible to cadmium toxicity and in the further life up to reaching adulthood. Moreover, the administration of ChE alone by 3 and 10 months allowed us to estimate the effect of its prolonged enhanced consumption on the young intensively growing organism and the adult one.

After 3 and 10 months of the experiment, the right and left sublingual salivary glands were collected under general anaesthesia with barbiturate (intraperitoneal administration of Morbital in a dose of 30 mg/kg b.w.), immediately after animals were sacrificed. For this purpose, the common sublingual and submandibular connective tissue capsule, covering both salivary glands, was removed, and the right and left sublingual glands (the sublingual glands are paired salivary glands of the same structure and function) were separated from the submandibular glands.

The dissected material (sublingual glands) was washed in ice-cold physiological saline (0.9% sodium chloride) and gently drained by using a filter paper. Next, the OHAUS® automatic balance (Nanikon, Switzerland) was used for weighing with accuracy to 0.0001 g. Finally, the material was frozen (–80°C).

### 2.2. Analytical Procedures

#### 2.2.1. Assay of Markers of Oxidative/Antioxidative Balance and Oxidative Stress

Because the sublingual salivary gland of a rat is very small (mean weight of the left and right glands together was about 0.08 g), both glands were used to perform all planned measurements. The half of the left salivary gland and the whole right one (of each rat) were homogenized in a cold 50 mM phosphate buffer (pH = 7.4) using an Ultra-Turrax T25 knife homogenizer (IKA, Staufen, Germany). Each of the obtained 10% homogenates was divided into two parts. One of the samples was centrifuged (MPW-350R, Medical Instruments, Warsaw, Poland) at 700 × *g* for 20 min at 4°C and used for the determination of GSH, CAT, LPO, TAS, TOS, H_2_O_2_, and PC. The second sample was centrifuged at 20000 × *g* for 30 min at 4°C and was assigned to assay SOD and GPx [50]. After centrifugation, the aliquots were separated and divided into portions. Next, they were frozen (–80°C) and stored in this state until the scheduled tests were performed.

The activity of CAT was measured according to Aebi based on the spectrophotometric measurement of the amounts of H_2_O_2_ (CHEMPUR, Piekary Śląskie, Poland) degraded by this enzyme [51]. The assay was performed with the precision, expressed as a coefficient of variation (CV), of <5.6%.

The activity of GPx (BIOXYTECH GPx-340™ kit) and the concentrations of H_2_O_2_ (BIOXYTECH H_2_O_2_-560™ kit) and LPO (BIOXYTECH LPO-586™ kit) were determined using diagnostic kits by Percipio Biosciences (Burlingame, CA, USA). The BIOXYTECH GPx-340™ kit measures the rate of reduction of oxidized glutathione to GSH that is mediated by GPx. The rule of the assay of H_2_O_2_ consists in oxidation by this compound of divalent iron to its trivalent form. The determination of LPO is based on the reaction of malondialdehyde and 4-hydroxyalkenal, present in an assayed sample, with N-methyl-2-phenylindole added to the sample and measurement of the absorbance of the product of the reaction at 586 nm. The precision of the methods was <3.2%, <4.0%, and<6%, respectively.

The Superoxide Dismutase Assay Kit and Glutathione Assay Kit by Cayman Chemical Company (Ann Arbor, MI, USA) were used for the determination of the activity of SOD and the concentration of GSH, respectively. The Superoxide Dismutase Assay Kit measures all three types of SOD (copper/zinc-SOD, manganese-SOD, and iron-SOD). A tetrazolium salt is utilized in the assay for the detection of superoxide radicals (O_2_^.−^) generated by hypoxanthine and xanthine oxidase. The amount of SOD needed for the dismutation of 50% of the generated O_2_^.−^ is defined as one unit of the enzyme activity. The assay of GSH is based on the reaction of GSH and 5,5′-dithio-bis-2-nitrobenzoic acid and measurement at 405–412 nm of the absorbance of a generated product. The CV for the assay of SOD and GSH was <4.5% and <3.4%, respectively.

The diagnostic ImAnOx (TAS) Kit and PerOx (TOS) Kit by Immundiagnostik AG (Bensheim, Germany) were used for the determination of TAS and TOS, respectively. The assay of TAS is based on the assay of the products of the reaction of residual H_2_O_2_ added into the sample. The added H_2_O_2_ is eliminated by antioxidants present in the assayed sample, whereas the residual H_2_O_2_ reacts with other compounds resulting in the generation of products showing absorbance at 450 nm. TOS determination is based on the measurement (at 450 nm) in the assayed sample of total lipid peroxides in the reaction with peroxidase. TAS values determined in the control samples (CTRL1—206.5 ± 4.950 *μ*mol/l and CTRL2—222.5 ± 9.192 *μ*mol/l; mean ± standard deviation (SD)) confirmed the correctness of the assay (values given by the producer CTRL1—170–230 *μ*mol/l and CTRL2—195–263 *μ*mol/l), and the precision (CV) was <4%. TOS values determined in the control samples (CTRL1—204.0 ± 8.485 *μ*mol/l and CTRL2—515.0 ± 21.23 *μ*mol/l) were within the limits given by the producer (CTRL1—185–308 *μ*mol/l and CTRL2—385–641 *μ*mol/l). The precision of the assay was <4.5%. To estimate the intensity of oxidative stress, the oxidative stress index (OSI) was calculated as the ratio of TOS and TAS.

The concentration of PC was measured spectrophotometrically by the method reported by Reznick and Packer consisting in the reaction of added 2,4-dinitrophenylhydrazine with PC present in a sample [52]. The precision of the assay was <6%.

In the supernatants of the homogenates of the sublingual gland tissue, total protein concentration was determined by the spectrophotometric method using a BioMaxima diagnostic kit (Lublin, Poland). The values of all parameters were normalized by protein concentration. The concentration of protein in the groups treated with cadmium and/or ChE and control animals did not differ (Table S5 in Supplementary Materials).

All these tests were performed following the producer's instructions, and particular parameters were determined in duplicate. The instruments used included an automatic microplate washer Wellwash 4 (Thermo Labsystems, Helsinki, Finland), a microplate reader Epoch (BioTek Instruments, Inc.; Winooski, USA), and a spectrophotometer UV-VIS SPECORD 50 PLUS (Analytik Jena, Jena, Germany).

#### 2.2.2. Cadmium Assay

The concentration of cadmium in the salivary gland tissue, after its wet digestion, was determined using the atomic absorption spectrometry (AAS) method with electrothermal atomization in the graphite cuvette (Pyro cuvette A, Hitachi). The HITACHI Z-5000 (Tokyo, Japan) atomic absorption spectrophotometer with a hollow cathode lamp (Photron, Narre Warren, Australia) for the detection of this element was used. The halves of the left sublingual salivary gland were digested with a trace-pure concentrated nitric acid using the UniClever II microwave system (Plazmatronika, Wroclaw, Poland). Next, the tissue digests were diluted with ultrapure water. The ultrapure water was received from the two-way water purification MAXIMA system by ELGA (Bucks, Great Britain). Cadmium concentration determined simultaneously in the analyzed reference material (bovine muscle, ERM-BB184, Geel, Belgium) (0.0023 ± 0.0001 *μ*g/g) agreed with the certified value (0.0022 *μ*g/g). The CV was <5%.

Based on cadmium concentration determined in the left sublingual salivary gland tissue, the total content of this element in this gland was calculated by multiplication of cadmium concentration (expressed as *μ*g/g of wet tissue weight) and this gland wet weight (expressed in grams). Moreover, to evaluate this toxic heavy metal total content in both salivary glands, it was assumed that its concentration in the left gland and the right one is on the same level.

### 2.3. Statistic

The results were statistically analyzed using the computer program Statistica 13 (StatSoft; Tulsa, USA). Because no normal distribution of the data was found (Shapiro-Wilk test), the results are presented as median and minimum and maximum for each group of 8 rats, and the nonparametric Kruskal-Wallis test was performed to check the occurrence of statistically significant (*p* < 0.05) differences between the experimental groups. In the case of detection of such differences, the Kruskal-Wallis signed-rank nonparametric test was performed to determine which two means differed statistically significantly (*p* < 0.05). To estimate the impact of cadmium and ChE administered alone or together on the values of particular parameters, the values in particular groups were compared to those in the control group. Moreover, to assess the influence of ChE coadministration under the exposure to cadmium, the values of particular parameters in the groups coadministered with cadmium and ChE were also compared to those in the respective groups treated with cadmium alone (Cd_1_+ChE vs. Cd_1_ and Cd_5_+ChE vs. Cd_5_). The independent and interactive effect of cadmium and ChE on the indices of the oxidative/antioxidative status of the sublingual salivary gland was assessed using a two-way analysis of variance (ANOVA/MANOVA, test *F*). *F* values with *p* < 0.05 were statistically significant. Pearson's correlation analysis was performed to evaluate the mutual relationships between the investigated markers describing the oxidative and antioxidative status of the sublingual salivary gland, as well as between these parameters and cadmium concentration and content in this gland. Correlations were recognized as statistically significant at the correlation coefficient (*r*) of *p* < 0.05.

## 3. Results

### 3.1. Effects of Cadmium and/or ChE on the Macroscopic Picture and Weight of the Sublingual Salivary Glands

All sublingual glands were of normal macroscopic structure. They were bright red and had a smooth surface. The left and right salivary glands were of the same weight (data not shown). There were no differences in the absolute and relative weights of the sublingual glands among the experimental groups at the studied time points (3 and 10 months), except for the Cd_1_+ChE group in which both weights after 10 months were higher than those in the control females (by 83% and 69%, respectively) and the animals administered with ChE alone (by 86% and 64%, respectively) (Table 1).

### 3.2. Effects of Cadmium and/or ChE on the Oxidative/Antioxidative Balance of the Sublingual Salivary Gland

**Figure 1:**
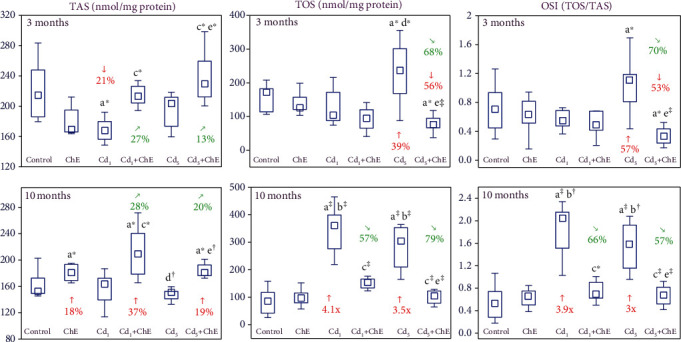
The effects of cadmium (Cd) and chokeberry extract (ChE) on the total antioxidative status (TAS), total oxidative status (TOS), and oxidative stress index (OSI) of the sublingual salivary gland of female rats. The females received cadmium in the amount of 1 and 5 mg Cd/kg feed and/or 0.1% ChE. Data are presented as median, 25–75% confidence interval, and minimum and maximum for eight rats in each group (in Figure S1 in Supplementary Materials, the data are presented as individual points for each of eight rats per group). Statistically significantly different (Kruskal-Wallis post hoc test) versus ^a^the control group, ^b^the ChE group, ^c^the Cd_1_ group, ^d^the Cd_1_+ChE group, and ^e^the Cd_5_ group, where ^∗^*p* < 0.05, ^†^*p* < 0.01, and ^‡^*p* < 0.001. Numerical values above or below the points presenting the median values reveal the percentage changes or factors of changes versus the respective control group (↓, decrease; ↑, increase) or the appropriate group that did not receive ChE during the treatment with cadmium (↘, decrease; ↗, increase).

The intake of ChE alone had no impact on the values of TAS, TOS, and OSI of the sublingual salivary gland, except for an increased (by 18%) TAS after its 10-month consumption (Figure 1).

The feeding with the 1 and 5 mg Cd/kg diet did not influence TAS of the salivary gland tissue except for its decrease (by 21%) due to the 3-month low-level exposure. Moreover, the treatment with cadmium increased the tissue TOS (by 39% to 4.1-fold) and OSI (by 57% to 3.9-fold), except for a lack of change of these parameters in the Cd_1_ group after 3 months (Figure 1).

The administration of ChE under the 3-month treatment with the 1 mg Cd/kg diet prevented this heavy metal-induced drop in the value of TAS. Moreover, the extract coadministration increased (by 13–28%) this heavy metal-unaffected value of this parameter in the Cd_1_+ChE group after 10 months and Cd_5_+ChE group after 3 and 10 months making it higher at both levels of the 10-month experiment duration than in the control females (by 37% and 19%, respectively) (Figure 1). Moreover, the coadministration of ChE under the exposure to the 1 and 5 mg Cd/kg diet completely prevented this heavy metal-induced increase in the values of TOS and OSI, making them after the 3-month exposure to the 5 mg Cd/kg diet even lower than in the control group (Figure 1). The two-way variance analysis has revealed that ChE impact on the determined markers of the oxidative/antioxidative balance in the sublingual salivary tissue resulted from its independent and/or interactive antagonistic action with cadmium (Table 2).

### 3.3. Impact of Cadmium and/or ChE on the Enzymatic Antioxidative Barrier and GSH Concentration in the Sublingual Salivary Gland

**Figure 2:**
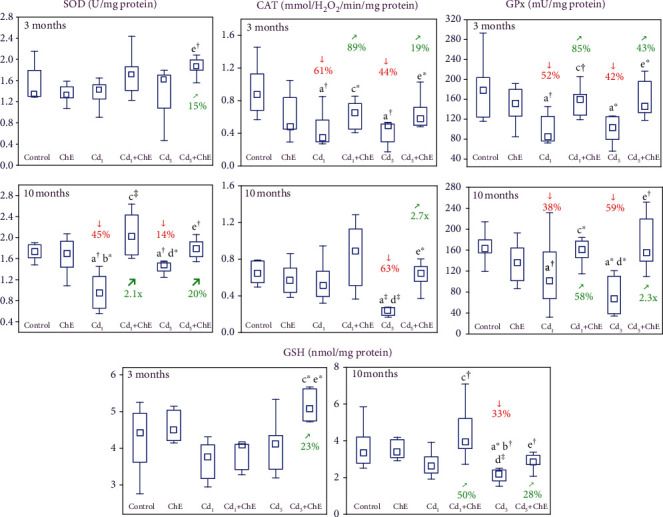
The effects of cadmium (Cd) and chokeberry extract (ChE) on the activities of superoxide dismutase (SOD), catalase (CAT), and glutathione peroxidase (GPx) and the concentration of reduced glutathione (GSH) in the sublingual salivary gland of female rats. The females received cadmium in the amount of 1 and 5 mg Cd/kg feed and/or 0.1% ChE. Data are presented as median, 25–75% confidence interval, and minimum and maximum for eight rats in each group (in Figure S2 in Supplementary Materials, the data are presented as individual points for each of eight rats per group). Statistically significantly different (Kruskal-Wallis post hoc test) versus ^a^the control group, ^b^the ChE group, ^c^the Cd_1_ group, ^d^the Cd_1_+ChE group, and ^e^the Cd_5_ group, where ^∗^*p* < 0.05, ^†^*p* < 0.01, and ^‡^*p* < 0.001. Numerical values above or below the points presenting the median values reveal the percentage changes or factors of changes versus the respective control group (↓, decrease) or the appropriate group that did not receive ChE during the treatment with cadmium (↗, increase).

The administration of ChE alone for 3 and 10 months had no effect on the activities of determined antioxidative enzymes (SOD, CAT, and GPx), as well as on the concentration of GSH in the sublingual salivary gland (Figure 2).

The activities of antioxidative enzymes in the sublingual salivary gland tissue were decreased (by 14–63%) in the Cd_1_ and Cd_5_ groups, except for SOD in both groups after 3 months and CAT in the former group after 10 months (Figure 2). The coadministration of ChE to the animals intoxicated with cadmium prevented this xenobiotic-induced drop in the activities of SOD, CAT, and GPx (Figure 2). According to the results of the two-way variance analysis, the impact of ChE on the enzymatic antioxidative barrier of the sublingual salivary gland was caused by its independent effect and/or interaction (antagonism) with cadmium (Table 3).

The concentration of GSH in the investigated salivary gland tissue was unaffected by cadmium except for its decrease (by 33%) after the 10-month maintenance on the diet contaminated with 5 mg Cd/kg (Figure 2). The effect of cadmium was completely counteracted by the coadministration of ChE (Figure 2); however, the two-way analysis of variance did not reveal an independent impact of ChE nor its interaction with cadmium (Table 3).

### 3.4. Impact of Cadmium and/or ChE on the Concentration of H_2_O_2_ in the Sublingual Salivary Gland

**Figure 3:**
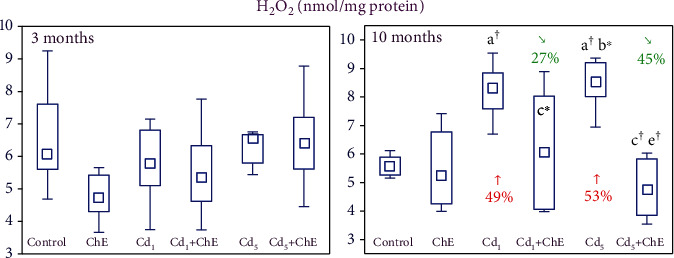
The effects of cadmium (Cd) and chokeberry extract (ChE) on the concentration of hydrogen peroxide (H_2_O_2_) in the sublingual salivary gland of female rats. The females received cadmium in the amount of 1 and 5 mg Cd/kg feed and/or 0.1% ChE. Data are presented as median, 25–75% confidence interval, and minimum and maximum for eight rats in each group (in Figure S3 in Supplementary Materials, the data are presented as individual points for each of eight rats per group). Statistically significantly different (Kruskal-Wallis post hoc test) versus ^a^the control group, ^b^the ChE group, ^c^the Cd_1_ group, and ^e^the Cd_5_ group, where ^∗^*p* < 0.05 and ^†^*p* < 0.01. Numerical values above or below the points presenting the median values reveal the percentage changes versus the respective control group (↑, increase) or the appropriate group that did not receive ChE during the treatment with cadmium (↘, decrease).

H_2_O_2_ concentration in the investigated salivary gland tissue of rats intoxicated with 1 and 5 mg Cd/kg of diet for 3 months was unchanged versus the control group, but after the 10-month exposure, the concentration increased (by 49% and 53%, respectively) (Figure 3).

The consumption of ChE alone did not influence the concentration of H_2_O_2_, whereas the extract application during the exposure to cadmium prevented this toxic element-caused elevation in the concentration of this marker of oxidative status (Figure 3). The protective effect of ChE at the conditions of the low-level cadmium treatment resulted from its interactive action with this xenobiotic but at the moderate exposure from both an interactive action of the extract ingredients with cadmium and their independent impact (Table 4).

### 3.5. Impact of Cadmium and/or ChE on LPO and PC Concentrations in the Sublingual Salivary Gland

**Figure 4:**
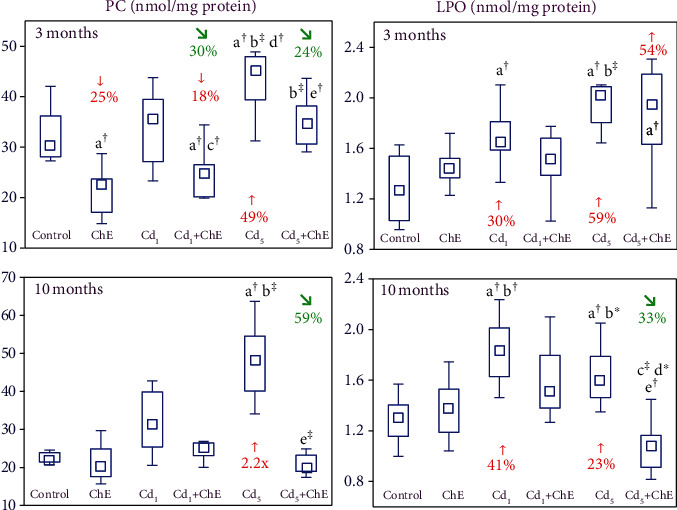
The effects of cadmium (Cd) and chokeberry extract (ChE) on the concentration of protein carbonyl groups (PC) and lipid peroxides (LPO) in the sublingual salivary gland of female rats. The females received cadmium in the amount of 1 and 5 mg Cd/kg feed and/or 0.1% ChE. Data are presented as median, 25–75% confidence interval, and minimum and maximum for eight rats in each group (in Figure S4 in Supplementary Materials, the data are presented as individual points for each of eight rats per group). Statistically significantly different (Kruskal-Wallis post hoc test) versus ^a^the control group, ^b^the ChE group, ^c^the Cd_1_ group, ^d^the Cd_1_+ChE group, and ^e^the Cd_5_ group, where ^∗^*p* < 0.05, ^†^*p* < 0.01, and ^‡^*p* < 0.001. Numerical values above or below the points presenting the median values reveal the percentage changes or factors of changes versus the respective control group (↑, increase; ↓, decrease) or the appropriate group that did not receive ChE during the treatment with cadmium (↘, decrease).

The up to 10-month supplementation with ChE alone had no impact on LPO and PC concentrations in the sublingual salivary gland, except for a reduction (by 25%) in the level of PC after 3 months (Figure 4).

Although the exposure to the 1 mg Cd/kg diet for 3 and 10 months did not influence the concentration of PC, it increased LPO concentration (by 30% and 41%, respectively) (Figure 4). In the animals maintained on the 5 mg Cd/kg diet, the levels of both indices of oxidative modifications of the cellular macromolecules were increased (by 23% to 2.2-fold) (Figure 4).

ChE coadministration under the low-level and moderate treatment with cadmium prevented this xenobiotic-caused elevation of PC and LPO concentrations in the sublingual salivary gland, except for LPO concentration after 3 months of the intoxication with the 5 mg Cd/kg diet (Figure 4). Moreover, the 3-month application of the extract to the animals maintained on the diet containing 1 mg Cd/kg decreased the cadmium alone-unchanged level of PC to the value lower (by 18%) than that in the control group (Figure 4). The favourable effect of ChE administration noted in the animals fed with the 1 and 5 mg Cd/kg diet for 3 months on the concentration of PC was caused by its independent action, whereas the impact under the 10-month treatment with the 5 mg Cd/kg diet resulted from the independent action of the extract ingredients and their antagonistic interaction with cadmium (Table 4). The two-way variance analysis disclosed no independent or interactive effects of ChE on LPO concentration in the Cd_1_+ChE group after 3 and 10 months although this parameter concentration in this group at both time points did not differ versus the control animals, while in the Cd_1_ group, it was higher (Figure 4). Moreover, the ANOVA/MANOVA confirmed no independent and interactive effects of ChE on LPO concentration in the Cd_5_+ChE group after 3 months (Figure 4).

### 3.6. Impact of ChE on Cadmium Accumulation in the Sublingual Salivary Gland

**Figure 5:**
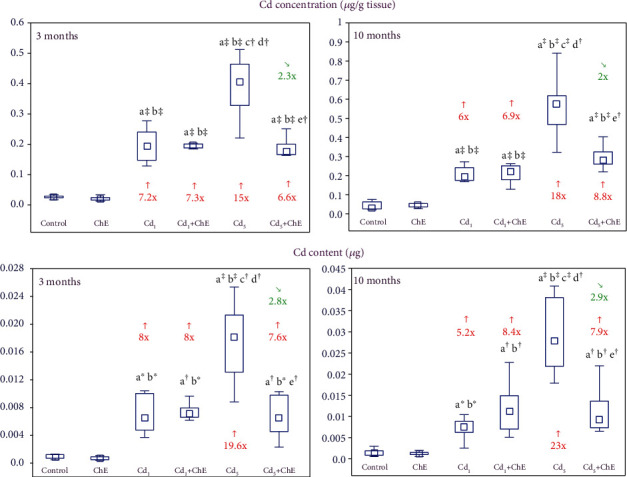
The effect of the chokeberry extract (ChE) on the concentration and content of cadmium (Cd) in the sublingual salivary glands of female rats exposed to this xenobiotic. The females received cadmium in the amount of 1 and 5 mg Cd/kg feed and/or 0.1% ChE. Data are presented as median, 25–75% confidence interval, and minimum and maximum for eight rats in each group (in Figure S5 in Supplementary Materials, the data are presented as individual points for each of eight rats per group). Statistically significantly different (Kruskal-Wallis post hoc test) versus ^a^the control group, ^b^the ChE group, ^c^the Cd_1_ group, ^d^the Cd_1_+ChE group, and ^e^the Cd_5_ group, where ^∗^*p* < 0.05, ^†^*p* < 0.01, and ^‡^*p* < 0.001. Numerical values above or below the points presenting the median values reveal the factors of changes versus the respective control group (↑, increase) or the appropriate group that did not receive ChE during the treatment with cadmium (↘, decrease).

The intake of ChE alone had no impact on the concentration of cadmium in the sublingual salivary gland tissue and the total content of this xenobiotic in these glands (Figure 5).

Cadmium exposure led to, dependent on its intensity, accumulation of this xenobiotic in the sublingual salivary glands as it was evident based on an increased concentration and total content of this heavy metal in these glands (Figure 5). This heavy metal concentration and content in the sublingual salivary glands of the animals fed with the 5 mg Cd/kg diet were higher 2.1- and 2.8-fold, respectively, after 3 months and 3- and 3.7-fold, respectively, after 10 months versus the female rats fed with the 1 mg Cd/kg diet.

There was no impact of ChE administration to the females maintained for 3 and 10 months on the diet containing 1 mg Cd/kg diet on this toxic element concentration and content in the sublingual salivary glands, while the extract coadministration during the moderate treatment with cadmium importantly protected against this toxic metal accumulation in these salivary glands.

Cadmium concentration and content in the Cd_5_+ChE group at particular time points were from 2- to 2.9-fold lower than those in the respective Cd_5_ group and within the ranges of values noted in the respective Cd_1_ group and Cd_1_+ChE group (Figure 5). The two-way variance analysis disclosed that the protective effect of the coadministration of ChE during the treatment with 5 mg Cd/kg diet on this xenobiotic accumulation in the sublingual salivary glands was caused by its components' independent and interactive action with cadmium (Table 5). The analysis also confirmed that cadmium concentration and its total content in the salivary glands in the Cd_1_+ChE were determined mainly by the treatment with this xenobiotic (Table 5).

### 3.7. Dependences between Cadmium Accumulation and the Investigated Markers of the Oxidative/Antioxidative Balance and Oxidative Stress of the Sublingual Salivary Gland

Numerous correlations occurred between the assayed indices of the oxidative/antioxidative balance and oxidative stress of the sublingual salivary gland (Table 6). Positive dependences were revealed between each two of the assayed markers of antioxidative status (GPx, CAT, GSH, SOD, and TAS), except for a lack of dependence between CAT and GSH), and oxidative status, except for no correlation between LPO and OSI (Table 6). TOS and OSI of the sublingual salivary gland tissue negatively correlated with GPx, GSH, SOD, and TAS (Table 6). Moreover, cadmium concentration and content in the sublingual salivary glands correlated negatively with some markers of the antioxidative status (CAT, GPx, and GSH) and positively with the markers of oxidative status (H_2_O_2_, TOS, OSI, and PC) (Table 6). Moreover, a positive dependence was noted between the content of cadmium in the sublingual salivary glands and LPO concentration (Table 6).

## 4. Discussion

The current study is the continuation and extension of our research conducted as a part of the project concerned with the protective effect of the chokeberry extract during the low and moderate (1 and 5 mg Cd/kg diet, respectively) exposure of rats to cadmium reflecting environmental human intoxication with this xenobiotic in industrialized countries. We confirmed the hypothesis that even low repeated exposure to cadmium may lead to destroying the oxidative/antioxidative balance and development of oxidative stress in the sublingual salivary gland, whereas the administration of ChE during the treatment may have a protective effect in this regard.

The oxidative-reductive processes in which ROS are produced and removed occur continuously in the body [3, 7, 8, 27, 28]. In normal conditions, the production and action of ROS and FR are balanced by the antioxidative barrier (both enzymatic and nonenzymatic), whose role consists in maintaining the equilibrium between the generation and removal of ROS and FR. A disorder in the balance may result in the development of oxidative stress due to an accumulation in cells of ROS, FR, and peroxides [2, 3]. ROS and FR can cause oxidative damage to all components of cells leading to irreversible injury to proteins, carbohydrates, and membranous lipids and modification of the chains of deoxyribonucleic acid (DNA) and ribonucleic acid (RNA) [26–28].

The performed determinations of the redox biomarkers in the present study allowed the estimation of the impact of cadmium and/or ChE on the balance between the processes of oxidation and reduction in the sublingual salivary gland tissue and the evaluation of the protective impact of the extract intake under cadmium intoxication regarding the prooxidative action of this toxic heavy metal. As expected, based on our previous results in these animals [7, 8, 26–28], the repeated treatment with the 1 and 5 mg Cd/kg diet destroyed the oxidative/reductive balance in the sublingual salivary gland leading to oxidative stress. Cadmium has a strong prooxidative potential and may negatively influence the oxidative/antioxidative status of various organs and tissues even at low concentrations [7, 8, 11, 26–28]. Although this xenobiotic cannot directly induce the formation of FR, ROS, or reactive nitrogen species (RNS), it can cause oxidative stress via indirect mechanism consisting in attenuating the antioxidative protection of the cells via weakening their enzymatic and nonenzymatic antioxidative barrier [7–9, 27, 28]. Moreover, this xenobiotic increases the release of ions of redox-active elements such as copper(I) and iron(II) (i.e., Cu^+^ and Fe^2+^) from the sites of their binding in the cells that directly increase the production of hydroxyl radicals (OH) in the Fenton reaction, damages the mitochondria, and induces the activity of oxidases [11, 31]. Based on the performed measurements, it may be concluded that the oxidative stress caused by cadmium in the sublingual salivary gland tissue resulted from the weakening of the enzymatic (decreased GPx, SOD, and CAT activities) and nonenzymatic (decreased GSH concentration) antioxidative protective barrier and enhanced amount of H_2_O_2_ indicating excessive accumulation of ROS. Both low-level and moderate cadmium intoxication led to oxidative modifications of macromolecules in the sublingual salivary gland; however, at the lower treatment, only lipid peroxidation was enhanced, whereas the higher intoxication also resulted in oxidative protein modifications. This finding indicates that lipids in the sublingual salivary gland tissue are more susceptible to oxidative modifications than proteins.

The current study not only confirmed our previous findings [7, 8] that even low intoxication with cadmium destroys the oxidative antioxidative balance and leads to the state of oxidative stress with modifications of the cellular macromolecules in the salivary gland tissue but also revealed that the effect of this xenobiotic on the balance between the processes of oxidation and reduction in the salivary glands differs dependent on the kind of these glands. Detailed comparative analysis of the findings of the present work regarding the sublingual salivary gland with the results on the submandibular [7] and parotid [8] salivary glands allowed for the conclusion that the sublingual gland may be the least susceptible to the development of the state of imbalance between the processes of oxidation and reduction and to oxidative changes. In the animals fed with the diet containing 1 mg Cd/kg, oxidative stress (recognized based on the calculation of OSI) in the submandibular salivary gland was noted already after 3 months [7]. In the parotid salivary gland [8], analogously as in the sublingual salivary gland, the value of OSI at the low-level treatment was enhanced after 10 months; however, the concentration of PC was increased already after 3 months. Moreover, it is worthy of underlining that in the sublingual and submandibular [7] salivary glands, unlike the parotid gland [8], lipid peroxidation was enhanced already after the 3-month intoxication with the 1 mg Cd/kg diet. The impact of cadmium on the balance between the processes of oxidation and reduction in the sublingual salivary gland has been the subject of very little experimental research so far [9, 53]. Only Kostecka-Sochoń et al. [9, 53] reported reduced activities of GPx (by 39% and 38%, respectively) and SOD (by 26% and 33%, respectively) and the concentration of GSH (by 24% and 29%, respectively) and unchanged activity of CAT under the conditions corresponding to moderate and relatively high (5 and 50 mg Cd/l, respectively) chronic human intoxication with this element. Moreover, the concentrations of H_2_O_2_ and LPO in these animals exceeded the values noted in the control ones (by 26% and 96% and 2.2- and 3-fold, respectively). However, only the current work is the first showing the prooxidative impact of cadmium on the sublingual salivary gland at low exposure, corresponding to the general population exposure in industrialized countries. Bittencourt et al. [5] reported increased concentration of malondialdehyde (MDA), reflecting enhancement in the process of oxidation of lipids, in the parotid, submandibular, and sublingual salivary gland tissue after 60-day oral rat intoxication with methylmercury in a daily dose of 0.04 mg/kg b.w.

The small weight of the sublingual salivary glands made it impossible to evaluate the microscopic structure of the gland tissue, but the fact that the low-level and moderate exposure to cadmium led to destroying the oxidative/antioxidative balance and enhanced lipid peroxidation and caused oxidative protein modifications allows supposing that this xenobiotic might also cause pathological changes in the morphology of the sublingual salivary gland tissue. The pathways of cytotoxic action of cadmium are multidirectional and very complex; however, oxidative stress is the main mechanism of this xenobiotic cytotoxicity [14, 23, 25, 30]. Cadmium ions (Cd^2+^) possess a high affinity for biological structures containing -SH groups such as GSH and cysteine resulting in disturbance of their proper functions, including inhibition of antioxidative enzymes and GSH depletion [23, 30]. Ions of cadmium interfere with ions of essential elements like calcium, magnesium, zinc, copper, iron, and selenium [23, 25, 30]. After entering the cells, Cd^2+^ ions simulate ions of bioelements to influence the activity of some enzymes (activation or inhibition) interfering in this way with the metabolism of the cells [14, 23, 30]. At the cellular level, mitochondria are the major target structure for toxic action of cadmium. By decreasing the potential of mitochondrial membranes, Cd^2+^ ions disrupt the oxidative phosphorylation and adenosine triphosphate (ATP) synthesis [14, 23, 30]. Cadmium-induced oxidative stress leads to oxidative damage to the key cellular macromolecules such as lipids, proteins, and DNA [7–9, 14, 15, 23, 26, 28]. The disturbed balance between oxidants and antioxidants results in oxidative damage to the lipids, including phospholipids and proteins in the cellular membranes altering their fluidity and permeability. Moreover, cadmium inhibits the monooxygenase system and lowers the concentration of cytochrome P450 as a result of stimulation of lipid peroxidation and induction of heme oxygenase, catalysing heme degradation [23]. Via the above-described mechanisms, this heavy metal affects the proliferation and differentiation of cells and may lead to their apoptosis or necrosis [23, 27].

The present study allowed concluding not only regarding the damaging impact of Cd on the salivary glands but also on the protective role of ChE in these glands. The extract administration to the animals intoxicated with cadmium improved the antioxidative capability of the sublingual salivary gland and prevented excessive retention of H_2_O_2_ in this tissue protecting in this way against the occurrence of the state of oxidative stress and its outcomes such as intensification of the process of peroxidation of lipids and oxidative protein modifications. The findings of statistical analysis (ANOVA/MANOVA) and our previously published results in these animals [7, 8, 26–28] allow us to conclude that the protective impact of the ChE on the balance between the processes of oxidation and reduction in the sublingual salivary gland may be explained by its independent action and the interaction of its ingredients with cadmium. The main effect (independent action) of ChE resulted from its high antioxidative properties, determined first of all by the high presence of polyphenolic compounds (mainly anthocyanins) [29, 37] and the presence of other substances having antioxidative properties, including vitamins E and C and elements such as selenium and zinc [29]. The polyphenolic content of the chokeberry extract used in our experiment was certified by the producer, and the phytochemical profile of the extract was also evaluated by us [37]. The powdered extract by the Adamed Consumer Healthcare Company contained 612.40 ± 3.33 mg of total polyphenols/g (mean ± standard error), and the concentration of these compounds in the 0.1% aqueous extract administered to the animals reached 0.612 ± 0.003 mg/ml. Anthocyanins (202.28 ± 1.28 mg/g), including cyanidin 3-O-*β*-galactoside (80.07 ± 1.05 mg/g), cyanidin 3-O-*α*-arabinoside (33.21 ± 0.01 mg/g), and cyanidin 3-O-*β*-glucoside (3.68 ± 0.01 mg/g), as well as proanthocyanidins (129.87 ± 1.12 mg/g), phenolic acids (110.92 ± 0.89 mg/g, including chlorogenic acid at the concentration of 68.32 ± 0.08 mg/g), and flavonoids (21.94 ± 0.98 mg/g), were identified [37]. The antioxidative action of chokeberries and their products involves the uptake of FR and inhibition of generation of ROS and RNS, as well as a favourable influence on the activities of antioxidative enzymes [29, 34, 36, 54].

Based on the study and our previous results [7, 8, 19], the mechanism of the interactive beneficial effect of ChE on the balance between the processes of oxidation and reduction in the sublingual salivary gland may be explained by interactions of the substances present in the extract with cadmium which result in the lower content of this xenobiotic in the body, including, as it was shown in the current paper, its lower amount in the salivary glands. Polyphenolic compounds, possessing –OH groups, as well as other ingredients of the extract, such as fibre and pectin, are capable of binding divalent cadmium ions (Cd^2+^), influencing, as a result, the body turnover of this toxic heavy metal [7, 8, 19]. When cadmium and ChE are administered orally, as it took place in the case of our study, the interactions between them may occur already in the alimentary tract. It has been already reported that in the animals receiving 0.1% ChE during exposure to cadmium, this element's absorption from the digestive tract was decreased, its excretion with the urine was increased, and accumulation in various organs and the blood concentration were lower [7, 8, 19].

The ANOVA/MANOVA disclosed that the positive outcome of the administration of ChE on the balance between oxidants and antioxidants in the sublingual salivary gland at both levels of exposure to cadmium was the main effect of the extract action and its components' antagonistic interaction with this xenobiotic. Moreover, the fact that the extract intake during the treatment with the 1 mg Cd/kg diet did not protect against this heavy metal retention in the salivary gland while its administration under the moderate intoxication (5 mg Cd/kg diet) importantly protected against cadmium accumulation seems to indicate that the beneficial effect of ChE at the low cadmium treatment might be mainly caused by the strong antioxidative properties of the extract. It is important to underline that the accumulation of cadmium in the salivary gland (estimated based on its concentration and content) of the rats receiving the extract and fed with the diet contaminated with 5 mg Cd/kg did not differ versus the animals treated with the 1 mg Cd/kg diet alone. These show that ChE administration under moderate exposure to cadmium may markedly protect against this xenobiotic accumulation in the sublingual salivary gland. The lower cadmium accumulation due to the extract administration determined its less harmful effect on this gland. It was reflected in the results of measurements of parameters reflecting the oxidative status and antioxidative status and the balance between them, including especially the lower values of TOS and OSI and the concentrations of PC, LPO, and H_2_O_2_, as well as in the higher antioxidative enzyme activities and the value of TAS in the Cd_5_+ChE group compared to the Cd_5_ group. The negative correlations noted between cadmium content and/or concentration in the sublingual salivary glands and some markers of the antioxidative barrier (GPx, CAT, and GSH), as well as the positive correlations between cadmium and the indices of oxidative status (TOS, OSI, H_2_O_2_, LPO, and PC), confirm the close relationship between this xenobiotic accumulation in these glands and their oxidative/antioxidative status. The noting of the improving impact of ChE intake during repeated exposure to cadmium on the oxidative/antioxidative balance of the sublingual salivary gland and the ability of the extract to prevent the occurrence of the state of oxidative stress and its consequences constitute the key and most important achievements of the current research being of practical importance.

The finding that ChE administration alone did not increase the antioxidative capacity of the sublingual salivary gland but importantly counteracted the ability of cadmium to induce oxidative stress shows that under the proper conditions, i.e., at the state of the balance between oxidants and antioxidants, the consumption of ChE will not have an impact on the antioxidative capacity of the sublingual salivary glands, but at the conditions of exposure to a factor decreasing antioxidative capacity, the extract will markedly improve the oxidative/antioxidative status even totally counteracting the prooxidative activity of this factor. The result revealing that ChE is capable of counteracting the prooxidative action of cadmium may indicate that consumption of chokeberry-based items will also provide effective protection against the effects of other xenobiotics characterized by prooxidative properties.

Apart from the studies conducted by our research team [7, 8], there is no data in the available literature on the effect of ChE on the balance between the oxidative and antioxidative status of the salivary glands during the low and moderate exposure to cadmium. However, some experiments demonstrated that polyphenols occurring in the whortleberry extract (anthocyanins, flavonoids, and phenolic acids) [38], red ginseng [39], and tea [40] attenuate oxidative changes in the salivary glands occurring under radiotherapy.

The study is of scientific value and has also practical implications; however, it was not possible to avoid some shortcomings. The main of them is that our findings on the beneficial influence of ChE are based on an experiment performed in female rats, and thus, the conclusions refer first of all to the salivary glands of females. Thus, it seems to be necessary to investigate in further study if the extract will also exert the protective impact in males. The next limitation is the small weight of the sublingual salivary glands, which made it impossible to perform a wider range of studies, including determination of indices of oxidative modifications of nucleic acids and evaluation of the microscopic structure of the salivary gland tissue. Another limitations of the study are the impossibility of explanation of the molecular pathways of the preventive action of ChE with respect to the oxidative stress and oxidative modifications of proteins and lipids induced by cadmium in the sublingual salivary gland, as well as whether the changes in the balance between the processes of oxidation and reduction in this salivary gland noted due to the administration of cadmium or/and ChE might be the outcome or the cause of the impact on the salivary glands' secretory function or saliva composition. In the available literature, we have found no data on the relationship between salivary glands' oxidative/reductive status and the saliva secreted by them. However, taking into account the available data [3], it can be assumed that the cadmium-induced destruction of the balance between the processes of oxidation and reduction in the salivary gland tissue may disturb the function of the salivary glands and modify the composition of saliva produced and secreted by them. Further study should include the evaluation of the molecular mechanisms explaining the obtained results and the dependence between the processes of oxidation and reduction in the salivary gland and the composition and quality of the secreted saliva.

## 5. Conclusions

The study has shown that low-level repeated intoxication with cadmium may cause the destruction in the oxidative/antioxidative balance in the sublingual salivary gland. Cadmium, dose, and duration dependently attenuated the antioxidative capacity (both enzymatic and nonenzymatic) and increased H_2_O_2_ concentration and TOS of the salivary gland resulting in the occurrence of the state of oxidative stress with oxidative modifications of lipids and proteins in the salivary gland. The application of the chokeberry extract under the treatment with cadmium prevented this xenobiotic-caused oxidative/reductive imbalance, resulting in an occurrence of oxidative stress with oxidative modifications of proteins and lipids in the sublingual salivary gland. The findings indicate that the intake of black chokeberry products during repeated exposure to cadmium can prevent the occurrence of oxidative stress and its outcomes in the sublingual salivary gland and thus counteract the unfavourable impact of this xenobiotic on the oral cavity. The results of the current paper have provided further evidence confirming that chokeberry products can effectively prevent against unfavourable impact of repeated exposure to cadmium on health.

## Figures and Tables

**Table 1:** Absolute and relative weights of the sublingual salivary glands in all experimental groups of female rats^1^.

Experimental group	3 months	10 months
Absolute weight of sublingual salivary glands × 10^−2^ (g), median (minimum–maximum)		
Control	6.2 (5.2–9.5)	6.5 (3.1–8.6)
ChE	6.5 (5.4–9.4)	6.4 (3.0–7.4)
Cd_1_	7.5 (3.9–9.4)	8.8 (4.6–10.9)
Cd_1_+ChE	7.5 (6.2–10.0)	11.9 (8.2–15.0)^a†b†^
Cd_5_	8.1 (6.7–10.2)	8.1 (4.7–10.9)
Cd_5_+ChE	8.2 (3.8–24.5)	8.0 (3.3–12.2)
Relative weight of sublingual salivary glands × 10^−2^, median (minimum–maximum)		
Control	2.02 (1.59–3.18)	1.46 (0.68–2.04)
ChE	2.26 (1.48–2.87)	1.51 (0.69–1.92)
Cd_1_	2.56 (1.26–3.08)	1.98 (1.06–2.61)
Cd_1_+ChE	2.56 (2.00–3.50)	2.47 (2.10–3.01)^a†b†^
Cd_5_	2.68 (2.15–3.51)	1.52 (1.01–2.50)
Cd_5_+ChE	2.58 (1.25–8.54)	1.99 (0.77–27.40)

^1^The absolute weight of the left and right salivary glands is presented. The relative weight of the sublingual salivary glands is expressed as the ratio of the weight of the two glands together (expressed as grams) and the body weight of rats (expressed as grams). Statistically significantly different (Kruskal-Wallis post hoc test) versus ^a^the control group and ^b^the ChE group. ^†^*p* < 0.01.

**Table 2:** The main and/or interactive effects (ANOVA/MANOVA) of cadmium (Cd) and chokeberry extract (ChE) on the total antioxidative status (TAS), total oxidative status (TOS), and oxidative stress index (OSI) of the sublingual salivary gland of female rats^1,2^.

Parameter	Duration (months)	1 mg Cd/kg diet+ChE				5 mg Cd/kg diet+ChE			
		Cd main effect	ChE main effect	Cd+ChE main effect	Possible character of Cd-ChE interaction	Cd main effect	ChE main effect	Cd+ChE main effect	Possible character of Cd-ChE interaction
TAS	3	NS	NS	9.275^†^	Antagonistic action 0 vs. –21^3^ + 0 0 vs. –21	NS	NS	5.802^∗^	Additive action 0 = 0 + 0
	10	NS	16.83^‡^	NS	No interaction	NS	29.76^‡^	NS	No interaction

TOS	3	—	—	—	—	NS	19.26^‡^	12.24^†^	Antagonistic action –56 vs. +39 + 0 –56 vs. +39
	10	76.78^‡^	25.43^‡^	34.58^‡^	Antagonistic action 0 vs. +4.1‐fold + 0 0 vs. +4.1-fold	32.38^‡^	23.21^‡^	32.16^‡^	Antagonistic action 0 vs. +3.5‐fold + 0 0 vs. +3.5-fold

OSI	3	—	—	—	—	NS	10.41^†^	8.779^†^	Antagonistic action –53 vs. +57 + 0 –53 vs. +57
	10	45.98^‡^	23.71^‡^	32.35^‡^	Antagonistic action 0 vs. +3.9‐fold + 0 0 vs. +3.9-fold	26.60^‡^	15.09^‡^	22.39^‡^	Antagonistic action 0 vs. +3‐fold + 0 0 vs. +3-fold

^1^The results are presented as *F* values and the level of statistical significance (*p*). *F* values having *p* < 0.05 were considered statistically significant (^∗^*p* < 0.05, ^†^*p* < 0.01, and ^‡^*p* < 0.001). NS—*p* > 0.05.^2^To estimate the possible character of Cd-ChE interaction, the effect noted at coadministration of cadmium and ChE was compared to the sum of the effects of cadmium and ChE action after their separate administration (Cd+ChE versus Cd effect+ChE effect). ^3^The values are percentage changes.

**Table 3:** The main and/or interactive effects (ANOVA/MANOVA) of cadmium (Cd) and chokeberry extract (ChE) on the enzymatic antioxidative barrier and the concentration of reduced glutathione (GSH) in the sublingual salivary gland of female rats^1,2^.

Parameter	Duration (months)	1 mg Cd/kg diet+ChE				5 mg Cd/kg diet+ChE			
		Cd main effect	ChE main effect	Cd+ChE main effect	Possible character of Cd-ChE interaction	Cd main effect	ChE main effect	Cd+ChE main effect	Possible character of Cd-ChE interaction
SOD	3	—	—	—	—	NS	NS	5.064^∗^	Additive action 0 = 0 + 0
	10	NS	19.56^‡^	25.11^‡^	Antagonistic action 0 vs. –45^3^ + 0 0 vs. –45	NS	NS	7.536^∗^	Antagonistic action 0 vs. –14 + 0 0 vs. –14

CAT	3	5.879^∗^	NS	NS	No interaction	6.620	NS	NS	No interaction
	10	—	—	—	—	13.37^†^	9.632^†^	20.44^‡^	Antagonistic action 0 vs. –63 + 0 0 vs. –63

GPx	3	6.472^∗^	NS	8.625^†^	Antagonistic action 0 vs. –52 + 0 0 vs. –52	NS	NS	6.218^∗^	Antagonistic action 0 vs. –42 + 0 0 vs. –42
	10	NS	NS	6.704^∗^	Antagonistic action 0 vs. –38 + 0 0 vs. –38	4.003^∗^	6.347^†^	22.72^‡^	Antagonistic action 0 vs. –59 + 0 0 vs. –59

GSH	3	—	—	—	—	NS	5.199^∗^	NS	No interaction
	10	NS	5.665^∗^	NS	No interaction	8.276^†^	NS	NS	No interaction

^1^The results are presented as *F* values and the level of statistical significance (*p*). *F* values having *p* < 0.05 were considered statistically significant (^∗^*p* < 0.05, ^†^*p* < 0.01, and ^‡^*p* < 0.001). NS—*p* > 0.05.^2^To estimate the possible character of Cd-ChE interaction, the effect noted at coadministration of cadmium and ChE was compared to the sum of the effects of cadmium and ChE action after their separate administration (Cd+ChE versus Cd effect+ChE effect). ^3^The values are percentage changes. GPx: glutathione peroxidase; SOD: superoxide dismutase; CAT: catalase.

**Table 4:** The main and/or interactive effects (ANOVA/MANOVA) of cadmium (Cd) and chokeberry extract (ChE) on the concentrations of hydrogen peroxide (H_2_O_2_), protein carbonyl groups (PC), and lipid peroxides (LPO) in the sublingual salivary gland of female rats^1,2^.

Parameter	Duration (months)	1 mg Cd/kg diet+ChE				5 mg Cd/kg diet+ChE			
		Cd main effect	ChE main effect	Cd+ChE main effect	Possible character of Cd-ChE interaction	Cd main effect	ChE main effect	Cd+ChE main effect	Possible character of Cd-ChE interaction
H_2_O_2_	3	—	—	—	—	—	—	—	—
	10	12.97^†^	NS	5.085^∗^	Antagonistic action 0 vs. +49^3^ + 0 0 vs. +49	10.79^†^	22.29^‡^	26.28^‡^	Antagonistic action 0 vs. +53 + 0 0 vs. +53

PC	3	NS	25.49^‡^	NS	No interaction	41.72^‡^	25.89^‡^	NS	No interaction
	10	—	—	—	—	35.37^‡^	41.40^‡^	34.98^‡^	Antagonistic action 0 vs. +2.2‐fold + 0 0 vs. +2.2-fold

LPO	3	NS	NS	NS	No interaction	15.28^‡^	NS	NS	No interaction
	10	19.51^‡^	NS	NS	No interaction	NS	9.662^†^	17.12^‡^	Antagonistic action 0 vs. +23 + 0 0 vs. +23

^1^The results are presented as *F* values and the level of statistical significance (*p*). *F* values having *p* < 0.05 were considered statistically significant (^∗^*p* < 0.05, ^†^*p* < 0.01, and ^‡^*p* < 0.001). NS—*p* > 0.05. ^2^To estimate the possible character of Cd-ChE interaction, the effect noted at coadministration of cadmium and ChE was compared to the sum of the effects of cadmium and ChE action after their separate administration (Cd+ChE versus Cd effect+ChE effect). ^3^The values are percentage changes.

**Table 5:** The main and/or interactive effects (ANOVA/MANOVA) of cadmium (Cd) and chokeberry extract (ChE) on the concentration and content of this heavy metal in the sublingual salivary gland of female rats^1,2^.

Level of exposure to Cd	Duration (months)	Cd main effect	ChE main effect	Cd+ChE main effect	Possible character of Cd-ChE interaction
Cd concentration					
1 mg Cd/kg diet+ChE	3	167.8^‡^	NS	NS	No interaction
	10	112.7^‡^	NS	NS	No interaction
5 mg Cd/kg diet+ChE	3	161.5^‡^	29.88^‡^	26.85^‡^	Antagonistic action 6.6-fold vs. +15‐fold + 0 6.6-fold vs. +15-fold
	10	171.1^‡^	19.83^‡^	21.74^‡^	Antagonistic action 8.8-fold vs. +18‐fold + 0 8.8-fold vs. +18-fold
Cd content					
1 mg Cd/kg diet+ChE	3	118.3^‡^	NS	NS	No interaction
	10	52.121^‡^	NS	4.278^∗^	Antagonistic action 8.4-fold vs. +5.2‐fold + 0 8.4-fold vs. +5.2-fold
5 mg Cd/kg diet+ChE	3	98.75^‡^	22.68^‡^	21.06^‡^	Antagonistic action 7.6-fold vs. +19.6‐fold + 0 7.6-fold vs. +19.6-fold
	10	53.33^‡^	14.50^‡^	13.99^‡^	Antagonistic action 7.9-fold vs. +23‐fold + 0 7.9-fold vs. +23-fold

^1^The results are presented as *F* values and the level of statistical (*p*). *F* values having *p* < 0.05 were considered statistically significant (^∗^*p* < 0.05, ^†^*p* < 0.01, and ^‡^*p* < 0.001). NS—*p* > 0.05. ^2^To estimate the possible character of Cd-ChE interaction, the effect noted at coadministration of cadmium and ChE was compared to the sum of the effects of cadmium and ChE action after their separate administration (Cd+ChE versus Cd effect+ChE effect).

**Table 6:** Mutual relationships (Pearson's correlation analysis) between the investigated markers of the oxidative/antioxidative balance and oxidative stress of the sublingual salivary gland, as well as between these parameters and cadmium (Cd) concentration and content in the salivary glands of female rats^1^.

Parameters	Markers of antioxidative status					Markers of oxidative status				
	GPx	SOD	CAT	GSH	TAS	H_2_O_2_	TOS	OSI	PC	LPO
SOD	0.330^†^	—								
CAT	0.353^‡^	0.255^∗^	—							
GSH	0.390^‡^	0.221^∗^	NS	—						
TAS	0.384^‡^	0.510^‡^	0.397^‡^	0.484^‡^	—					
H_2_O_2_	NS	NS	–0.197^∗^	NS	NS					
TOS	–0.275^†^	–0.378^‡^	NS	–0.299^†^	–0.335^‡^	0.577^‡^	—			
OSI	–0.331^†^	–0.374^‡^	NS	–0.360^‡^	–0.330^†^	0.525^‡^	0.849^‡^	—		
PC	–0.240^∗^	NS	–0.213^∗^	NS	NS	0.522^‡^	0.482^‡^	0.329^†^	—	
LPO	NS	NS	NS	NS	NS	0.447^‡^	0.341^‡^	NS	0.578^‡^	—
Cd concentration	–0.283^†^	NS	–0.353^‡^	–0.313^†^	NS	0.279^†^	0.349^‡^	0.328^†^	0.512^‡^	NS
Cd content	–0.350^‡^	NS	–0.391^‡^	–0.295^†^	NS	0.354^‡^	0.358^‡^	0.336^‡^	0.518^‡^	0.223^∗^

^1^The results are expressed as *r* values and the level of statistical significance (*p*). The values of *r* with *p* < 0.05 were considered statistically significant, where ^∗^*p* < 0.05, ^†^*p* < 0.01, and ^‡^*p* < 0.001. NS—lack of correlation (*p* > 0.05). GPx: glutathione peroxidase; SOD: superoxide dismutase; CAT: catalase; GSH: reduced glutathione; TAS: total antioxidative status; PC: protein carbonyl groups; LPO: lipid peroxides: H_2_O_2_: hydrogen peroxide; TOS: total oxidative status; OSI: oxidative stress index.

## Data Availability

The data used to support the findings of this study are available from the corresponding author upon request.

## Abbreviations

AAS method: Atomic absorption spectrometry
ANOVA/MANOVA: A two-way analysis of variance
b.w.: Body weight
CAT: Catalase
Cd: Cadmium
CV: Coefficient of variation
ChE: Extract from chokeberries (the berries of *Aronia melanocarpa* L.)
FR: Free radicals
GPx: Glutathione peroxidase
GSH: Reduced glutathione
H_2_O_2_: Hydrogen peroxide
LPO: Lipid peroxides
–OH group: Hydroxyl group
OSI: Oxidative stress index
O_2_^.−^: Superoxide radicals
*p*: Level of statistical significance
PC: Protein carbonyl groups
*r*: Correlation coefficient
RNS: Reactive nitrogen species
ROS: Reactive oxygen species
SOD: Superoxide dismutase
TAS: Total antioxidative status
TOS: Total oxidative status.

## Supplementary Materials

Supplementary Materials Table S1: the content of nutrients in the Labofeed diet. Table S2: the daily intake of food and drinking fluid in all experimental groups of rats. Table S3: the daily intake of cadmium (Cd) in all experimental groups of rats. Table S4: the daily intake of the extract from black chokeberries (ChE) and polyphenolic compounds in the experimental groups of rats administered with the extract alone and during the exposure to cadmium (Cd). Table S5: the concentration of total protein in the aliquots of 10% homogenates of the sublingual salivary gland tissue in all experimental groups of rats. Figure S1: the effects of cadmium (Cd) and chokeberry extract (ChE) on the total antioxidative status (TAS), total oxidative status (TOS), and oxidative stress index (OSI) of the sublingual salivary gland of female rats. Figure S2: the effects of cadmium (Cd) and chokeberry extract (ChE) on the activities of superoxide dismutase (SOD), catalase (CAT), and glutathione peroxidase (GPx) and the concentration of reduced glutathione (GSH) in the sublingual salivary gland of female rats. Figure S3: the effects of cadmium (Cd) and chokeberry extract (ChE) on the concentration of hydrogen peroxide (H_2_O_2_) in the sublingual salivary gland of female rats. Figure S4: the effects of cadmium (Cd) and chokeberry extract (ChE) on the concentration of protein carbonyl groups (PC) and lipid peroxides (LPO) in the sublingual salivary gland of female rats. Figure S5: the effect of chokeberry extract (ChE) on the concentration and content of cadmium (Cd) in the sublingual salivary glands of female rats exposed to this xenobiotic.
