# Supplementary material for: The Beneficial Impact of the Black Chokeberry Extract against the Oxidative Stress in the Sublingual Salivary Gland of Rats Intoxicated with Cadmium
Source: Oxid Med Cell Longev. 2021 Dec 31;2021:6622245. doi: 10.1155/2021/6622245 (PMC8741350; doi:10.1155/2021/6622245)
Supplement: Supplementary Materials — Table S1: the content of nutrients in the Labofeed diet. Table S2: the daily intake of food and drinking fluid in all experimental groups of rats. Table S3: the daily intake of cadmium (Cd) in all experimental groups of rats. Table S4: the daily intake of the extract from black chokeberries (ChE) and polyphenolic compounds in the experimental groups of rats administered with the extract alone and during the exposure to cadmium (Cd). Table S5: the concentration of total protein in the aliquots of 10% homogenates of the sublingual salivary gland tissue in all experimental groups of rats. Figure S1: the effects of cadmium (Cd) and chokeberry extract (ChE) on the total antioxidative status (TAS), total oxidative status (TOS), and oxidative stress index (OSI) of the sublingual salivary gland of female rats. Figure S2: the effects of cadmium (Cd) and chokeberry extract (ChE) on the activities of superoxide dismutase (SOD), catalase (CAT), and glutathione peroxidase (GPx) and the concentration of reduced glutathione (GSH) in the sublingual salivary gland of female rats. Figure S3: the effects of cadmium (Cd) and chokeberry extract (ChE) on the concentration of hydrogen peroxide (H2O2) in the sublingual salivary gland of female rats. Figure S4: the effects of cadmium (Cd) and chokeberry extract (ChE) on the concentration of protein carbonyl groups (PC) and lipid peroxides (LPO) in the sublingual salivary gland of female rats. Figure S5: the effect of chokeberry extract (ChE) on the concentration and content of cadmium (Cd) in the sublingual salivary glands of female rats exposed to this xenobiotic. [file 6622245.f1.docx]

# Oxidative Medicine and Cellular Longevity

**Supplementary Materials**

# The Beneficial Impact of Black Chokeberry Extract Against the Oxidative Stress in the Sublingual Salivary Gland of Rats Intoxicated with Cadmium

Barbara M. Onopiuk,^1^ Zofia N. Dąbrowska,^2^ Joanna Rogalska,^3^ Malgorzata M. Brzóska,^3^ Adam Dąbrowski,^1^ Kamil Bijowski,^4^ Pawel Onopiuk,^5^ Barbara Mroczko,^6^ Karolina Orywal, ^6^ Ewa Dąbrowska ^7^

^1^ Private Dental Office in Bialystok, Bialystok 15-773, Poland

^2^ Department of Periodontal and Oral Mucosa Diseases, Medical University of Bialystok,

Bialystok 15-276, Poland

^3^ Department of Toxicology, Medical University of Bialystok, Bialystok 15-222, Poland

^4^ Department of Maxillofacial and Plastic Surgery, Medical University of Bialystok, Bialystok

15-276, Poland

^5^ Department of Otholaryngology, Medical University of Bialystok, Bialystok 15-276, Poland

^6^ Department of Biochemical Diagnostics, Medical University of Bialystok, Bialystok 15-269,

Poland

^7^ Department of Gerostomatology, Medical University of Bialystok, Bialystok 15-286, Poland

Correspondence should be addressed to Zofia N. Dąbrowska; zofia.dabrowska@umb.edu.pl

## Table S1: The content of nutrients in the Labofeed diet. ^1^

| Balanced ingredient | | Content in 1 kg of the Labofeed diet | |
| --- | --- | --- | --- |
|  |  | Labofeed H | Labofeed B |
| Net energy | kcal/kg | 3 100 | 2 900 |
| General protein | % | 22 | 17 |
| Digestible protein | % | 19 | 15.5 |
| Crude fibre | % | 5 | 7 |
| Raw fat | % | 4 | 3.5 |
| Lysine | % | 1.5 | 1 |
| Methionine + cysteine | % | 0.8 | 0.65 |
| Tryptophan | % | 0.3 | 0.22 |
| Total calcium | % | 1.1 | 1 |
| Total phosphorus | % | 0.8 | 0.75 |
| Total sodium | % | 0.3 | 0.22 |

^1^ according to the producer (Animal Feed Manufacturer “Morawski”, Kcynia, Lublin, Poland)

## Table S2: The daily intake of food and drinking fluid in all experimental groups of rats ^1^.

| Group | Daily food intake [g/24 h/rat]  mean ± SE (range) | |  | Daily drinking fluid intake [ml/24 h/rat]  mean ± SE (range) | |
| --- | --- | --- | --- | --- | --- |
|  | 3 months | 10 months |  | 3 months | 10 months |
| Control | 24.51 ± 0.349  (22.6–25.9) | 20.09 ± 0.447  (18.4–22.6) |  | 43.1 ± 0.426  (41.0–45.0) | 41.16 ± 1.071  (36.0–45.5) |
| ChE | 24.42 ± 0.342  (22.9–26.2) | 21.71 ± 0.464  (19.0–23.0) |  | 43.20 ± 0.587  (40.7–45.3) | 40.93 ± 1.225  (34.0–46.5) |
| Cd_1_ | 22.94 ± 0.374  (20.7–24.8) | 20.52 ± 0.283  (19.4–22.1) |  | 43.03 ± 0.565  (40.0–45.0) | 43.17 ± 0.965  (38.5–47.7) |
| Cd_1_ + ChE | 23.55 ± 0.309  (21.7–24.7) | 20.8 ± 0.374  (18.9–22.8) |  | 43.14 ± 0.693  (39.1–46.3) | 41.06 ± 0.718  (37.5–43.7) |
| Cd_5_ | 24.17 ± 0.266  (23.2–25.5) | 20.89 ± 0.226  (19.7–22.1) |  | 43.0 ± 0.796  (39.5–45.7) | 41.87 ± 1.521  (32.7–47.0) |
| Cd_5_ + ChE | 24.39 ± 0.310  (22.1–25.4) | 20.06 ± 0.119  (19.6–20.8) |  | 42.82 ± 0.583  (39.7–46.0) | 42.40 ± 1.155  (35.9–46.3) |

^1^ The data on the daily intake of food and drinking fluid in the experimental model have already been published [19].

## Table S3: The daily intake of cadmium (Cd) in all experimental groups of rats ^1^.

| Group | Daily intake of Cd [μg/kg b.w.]  mean ± SE (range) | |
| --- | --- | --- |
|  | 3 months | 10 months |
| Control | 4.709 ± 0.067  (4.342–4.976) | 2.716 ± 0.060  (2.487–3.055) |
| ChE | 4.615 ± 0.065  (4.328–4.952) | 2.935 ± 0.063  (2.569–3.109) |
| Cd_1_ | 77.50 ± 1.264^‡^  (69.93–83.88) | 43.85 ± 0.60^‡^  (41.45–47.22) |
| Cd_1_ + ChE | 80.93 ± 1.06^‡^  (74.57–84.88) | 46.90 ± 0.84^‡^  (42.62–51.41) |
| Cd_5_ | 383.65 ± 4.44^‡^  (368.25–404.76) | 235.78 ± 2.55^‡^  (222.3 –249.44) |
| Cd_5_ + ChE | 385.92 ± 4.91^‡^  (349.68–401.8) | 239.66 ± 1.43^‡^  (234.17–248.51) |

^1^ Data represent the mean daily intake of cadmium ± standard error (SE) and its range throughout the 3- and 10-month study. Cadmium intake in the control group and group receiving the extract from black chokeberries (ChE) alone was calculated based on this xenobiotic concentration detected in the standard Labofeed diet (0.0584 ± 0.0049 mg/kg) [7, 19], while cadmium intake in the groups exposed to this element was calculated based on its concentration in the diet, declared by the producer (1 or 5 mg Cd/kg). ^‡^ *p* < 0.001 compared to the control group (nonparametric Kruskal–Wallis test).

## Table S4: The daily intake of the extract from black chokeberries (ChE) and polyphenolic compounds in the experimental groups of rats administered with the extract alone and during the exposure to cadmium (Cd) ^1^.

| Group | Daily intake of ChE ^1^ [mg/kg b.w.]  mean ± SE (range) | |  | Daily intake of polyphenols ^1^ [mg/kg b.w.]  mean ± SE (range) | |
| --- | --- | --- | --- | --- | --- |
|  | 3 months | 10 months |  | 3 months | 10 months |
| ChE | 139.22 ± 1.71  (131.72–146.60) | 94.73 ± 2.84  (78.70–107.64) |  | 91.53 ± 1.12  (86.6–96.4) | 62.29 ± 1.87  (51.7–70.8) |
| Cd_1_ + ChE | 148.26 ± 2.20  (129.90–153.82) | 92.67 ± 1.63  (84.55–98.53) |  | 97.47 ± 1.45  (88.3–104.6) | 60.92 ± 1.07  (55.6–64.8) |
| Cd_5_ + ChE | 140.58 ± 1.96  (125.63–145.57) | 99.08 ± 2.70  (85.68–110.50) |  | 92.41 ± 1.30  (85.3–98.8) | 65.14 ± 1.77  (55.1–71.1) |

^1^ Data represent the mean daily intakes of ChE or polyphenols ± standard error (SE) and their ranges throughout the 3- and 10-month study. The intake of polyphenols was calculated assuming that that the commercial extract contained 65.74% of these compounds (certified value). The intake of ChE and polyphenols in the control group, Cd_1_ group, and Cd_5_ group was assumed to be 0. There were no differences in ChE and polyphenols intake dependent on whteher the extract was administered alone or together with Cd (nonparametric Kruskal–Wallis test).

## Table S5: The concentration of total protein in the aliquots of 10% homogenates of the sublingual salivary gland tissue in all experimental groups of rats. ^1^

| Group | Protein concentration [g/l]  median (range) | |
| --- | --- | --- |
|  | 3 months | 10 months |
| Control | 2.097  (1.741–2.334) | 2.598  (2.201–22.6) |
| ChE | 2.391  (1.851–2.586) | 2.514  (2.006 ± 3.333) |
| Cd_1_ | 2.302  (2.034–2.615) | 2.187  (1.931–2.448) |
| Cd_1_ + ChE | 1.917  (1.787–2.443) | 2.289  (1.743–2.536) |
| Cd_5_ | 2.031  (1.840–2.124) | 2.198  (1.948–2.454) |
| Cd_5_ + ChE | 2.034  (1.572–2.345) | 2.603^c* e*^  (2.155–2.994) |

^1^ The animals received cadmium (Cd) in the diet at the concentrations of 1 and 5 mg Cd/kg and/or 0.1% aqueous extract from black chokeberries (ChE). Data are presented as median and range for eight rats in each group. Statistically significant differences (Kruskal-Wallis post hoc test): ^c^ compared to the Cd_1_ group, ^e^ compared to the Cd_5_ group, where * *p* < 0.05.


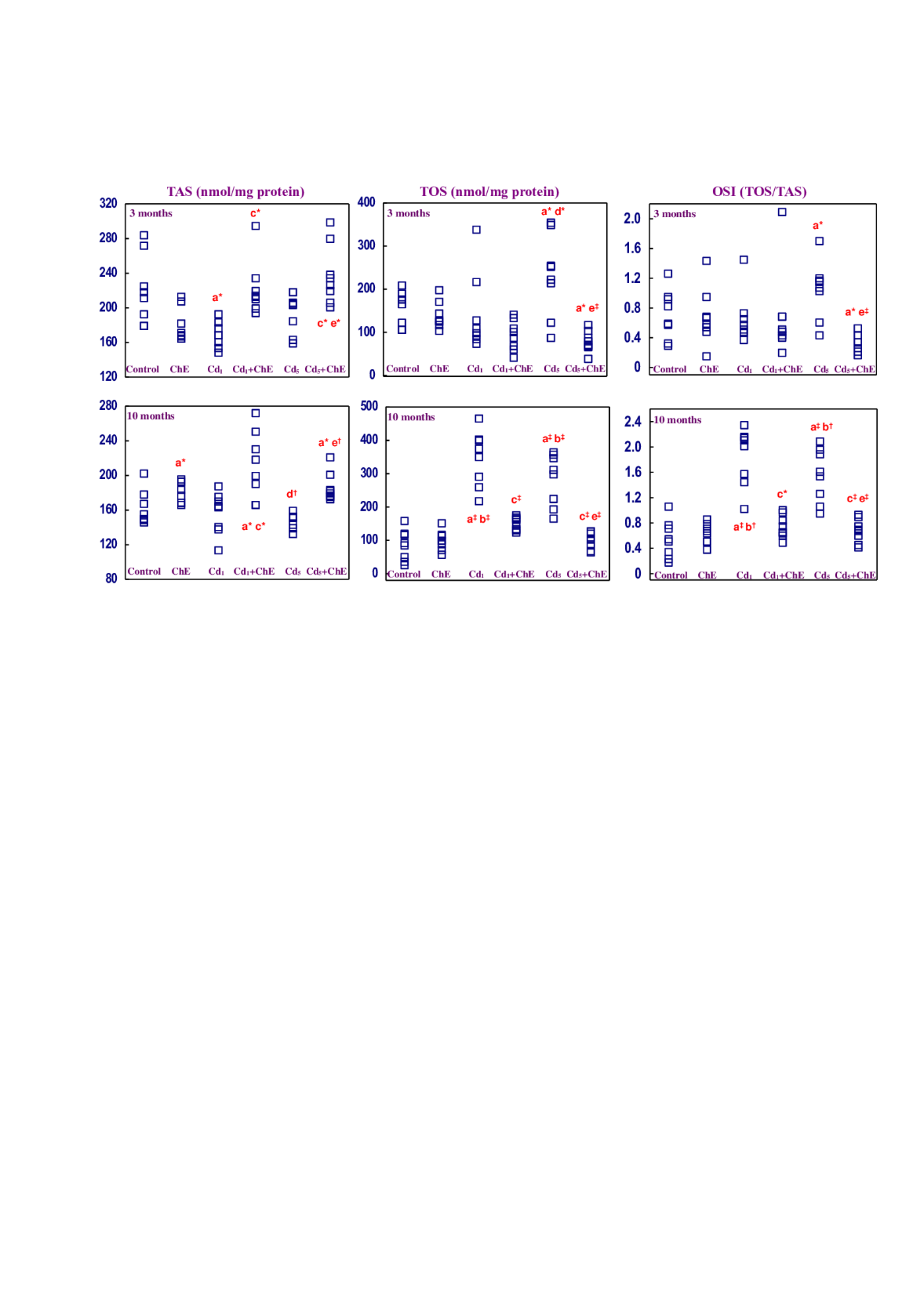


Figure S1: The effects of cadmium (Cd) and chokeberries extract (ChE) on the total antioxidative status (TAS), total oxidative status (TOS), and oxidative stress index (OSI) of the sublingual salivary gland of female rats. The females received cadmium in the amount of 1 and 5 mg Cd/kg feed and/or 0.1% ChE. Data are presented as individual points for each of eight rats per group. Statistically significantly different (Kruskal-Wallis post hoc test) versus: ^a^ the control group, ^b^ the ChE group, ^c^ the Cd_1_ group, ^d^ the Cd_1_ + ChE group, ^e^ the Cd_5_ group, where * *p* < 0.05, ^†^ *p* < 0.01, ^‡^ *p* < 0.001.


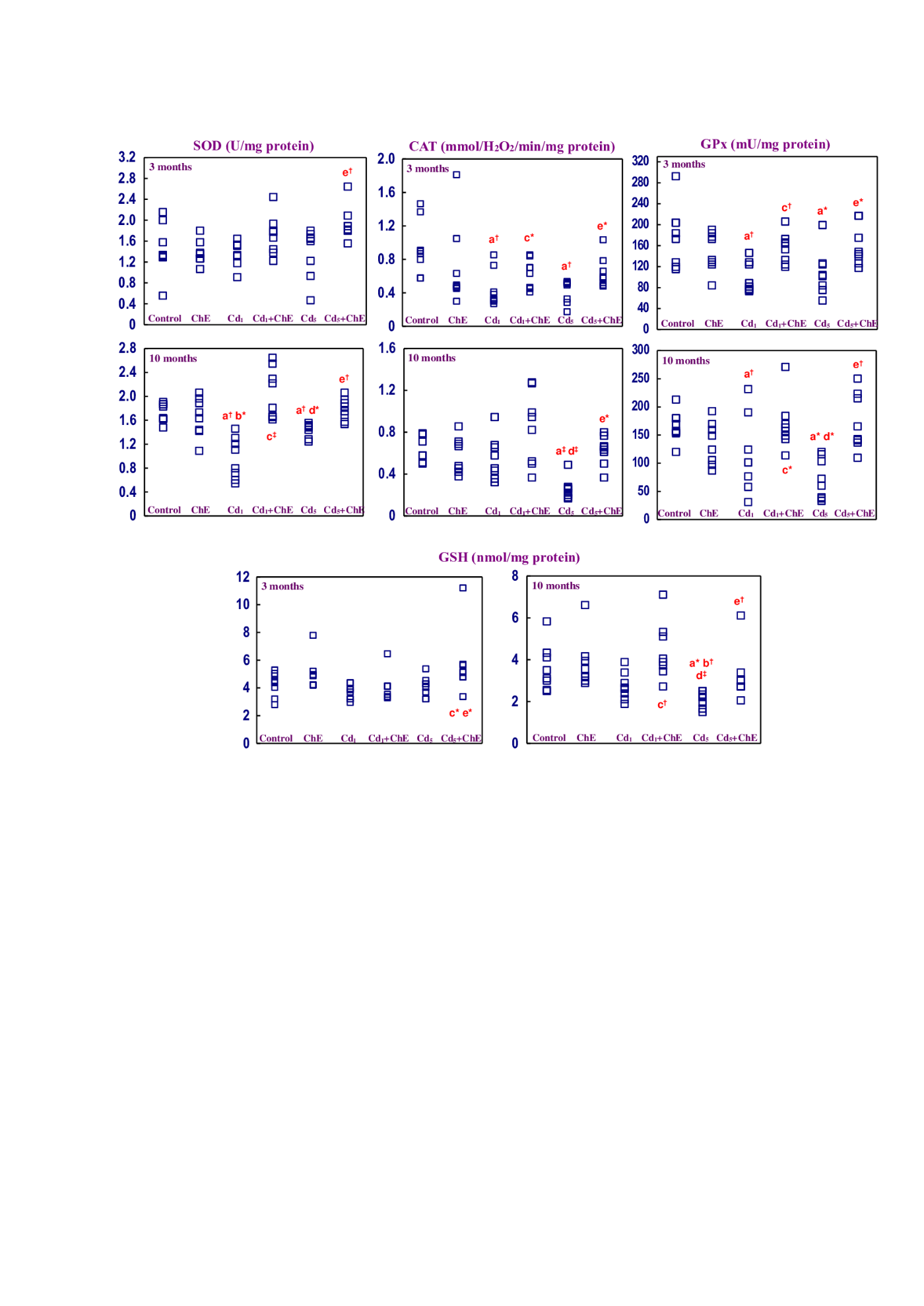


Figure S2: The effects of cadmium (Cd) and chokeberries extract (ChE) on the activities of superoxide dismutase (SOD), catalase (CAT), and glutathione peroxidase (GPx) and the concentration of reduced glutathione (GSH) in the sublingual salivary gland of female rats. The females received cadmium in the amount of 1 and 5 mg Cd/kg feed and/or 0.1% ChE. Data are presented as individual points for each of eight rats per group. Statistically significantly different (Kruskal-Wallis post hoc test) versus: ^a^ the control group, ^b^ the ChE group, ^c^ the Cd_1_ group, ^d^ the Cd_1_ + ChE group, ^e^ the Cd_5_ group, where * *p* < 0.05, ^†^ *p* < 0.01, ^‡^ *p* < 0.001.


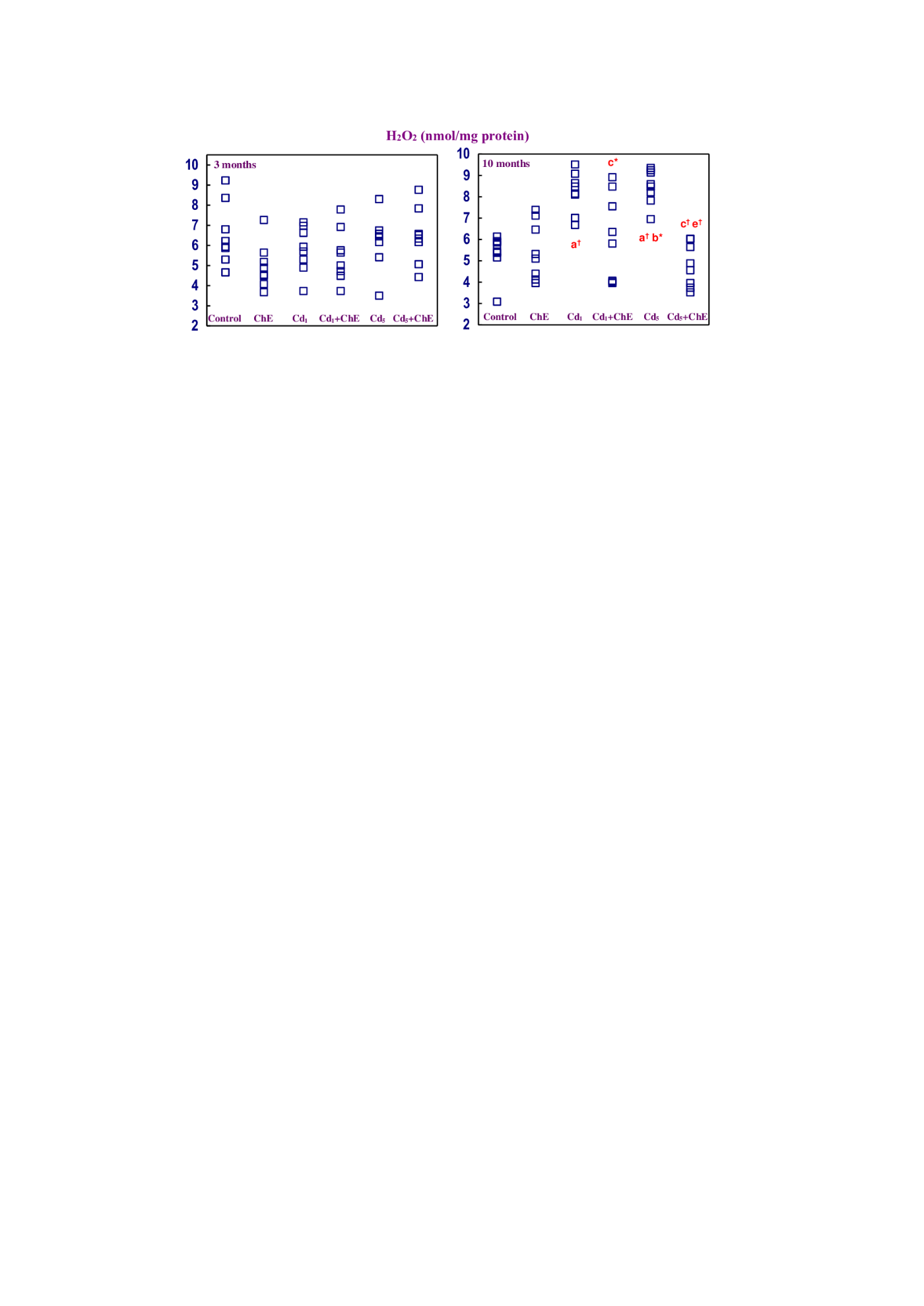


Figure S3: The effects of cadmium (Cd) and chokeberries extract (ChE) on the concentration of hydrogen peroxide (H_2_O_2_) in the sublingual salivary gland of female rats. The females received cadmium in the amount of 1 and 5 mg Cd/kg feed and/or 0.1% ChE. Data are presented as individual points for each of eight rats per group. Statistically significantly different (Kruskal-Wallis post hoc test) versus: ^a^ the control group, ^b^ the ChE group, ^c^ the Cd_1_ group, ^e^ the Cd_5_ group, where * *p* < 0.05, ^†^ *p* < 0.01.


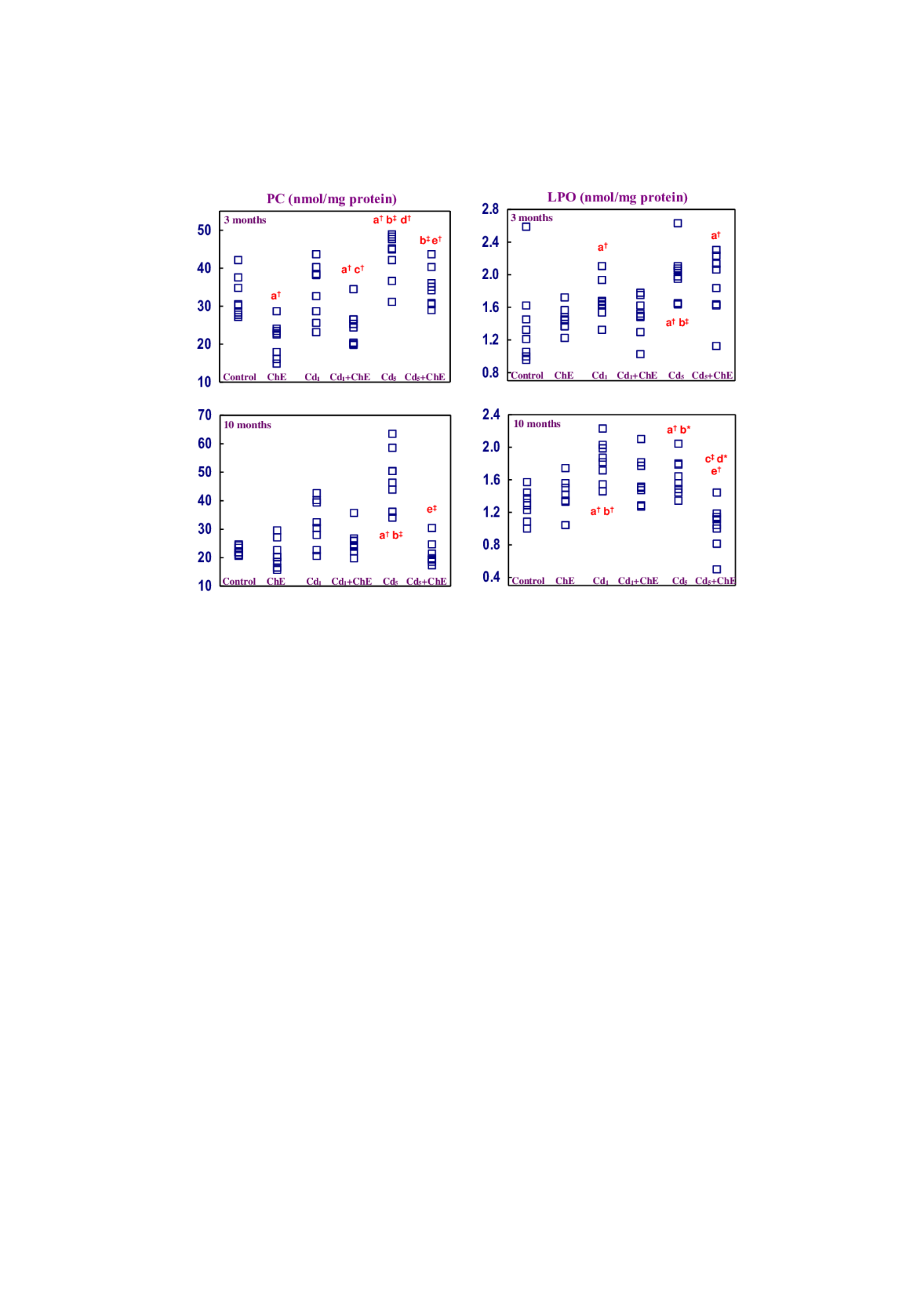


Figure S4: The effects of cadmium (Cd) and chokeberries extract (ChE) on the concentration of protein carbonyl groups (PC) and lipid peroxides (LPO) in the sublingual salivary gland of female rats. The females received cadmium in the amount of 1 and 5 mg Cd/kg feed and/or 0.1% ChE. Data are presented as individual points for each of eight rats per group. Statistically significantly different (Kruskal-Wallis post hoc test) versus: ^a^ the control group, ^b^ the ChE group, ^c^ the Cd_1_ group, ^d^ the Cd_1_ + ChE group, ^e^ the Cd_5_ group, where * *p* < 0.05, ^†^ *p* < 0.01, ^‡^ *p* < 0.001.


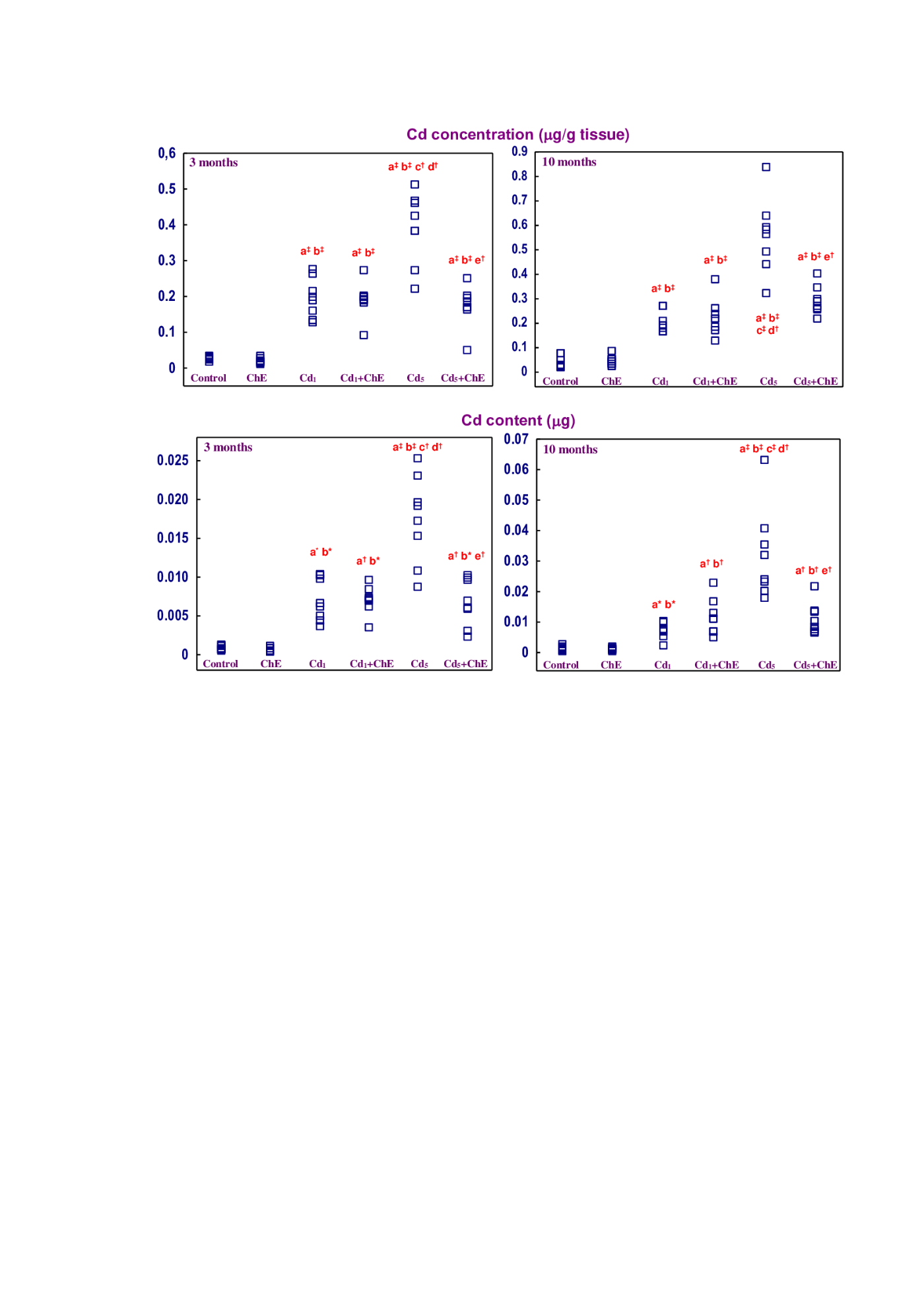


Figure S5: The effect of chokeberries extract (ChE) on the concentration and content of cadmium (Cd) in the sublingual salivary glands of female rats exposed to this xenobiotic. The females received cadmium in the amount of 1 and 5 mg Cd/kg feed and/or 0.1% ChE. Data are presented as individual points for each of eight rats per group. Statistically significantly different (Kruskal-Wallis post hoc test) versus: ^a^ the control group, ^b^ the ChE group, ^c^ the Cd_1_ group, ^d^ the Cd_1_ + ChE group, ^e^ the Cd_5_ group, where * p < 0.05, ^†^ p < 0.01, ^‡^ p < 0.001.
